# Minimally invasive detection of cancer using metabolic changes in tumor-associated natural killer cells with Oncoimmune probes

**DOI:** 10.1038/s41467-022-32308-x

**Published:** 2022-08-04

**Authors:** Deeptha Ishwar, Rupa Haldavnekar, Krishnan Venkatakrishnan, Bo Tan

**Affiliations:** 1 Institute for Biomedical Engineering, Science and Technology (I BEST), Partnership between Ryerson University and St. Michael’s Hospital, Toronto, ON M5B 1W8 Canada; 2 Ultrashort Laser Nanomanufacturing Research Facility, Faculty of Engineering and Architectural Sciences, Ryerson University, 350 Victoria Street, Toronto, ON M5B 2K3 Canada; 3 Nano Characterization Laboratory, Faculty of Engineering and Architectural Sciences, Ryerson University, 350 Victoria Street, Toronto, ON M5B 2K3 Canada; 4 Nano-Bio Interface facility, Faculty of Engineering and Architectural Sciences, Ryerson University, 350 Victoria Street, Toronto, ON M5B 2K3 Canada; 5 Keenan Research Center for Biomedical Science, Unity Health Toronto, Toronto, ON M5B 1W8 Canada

**Keywords:** Biomaterials - cells, Sensors and biosensors, Cancer stem cells, Neutrophils

## Abstract

Natural Killer (NK) cells, a subset of innate immune cells, undergo cancer-specific changes during tumor progression. Therefore, tracking NK cell activity in circulation has potential for cancer diagnosis. Identification of tumor associated NK cells remains a challenge as most of the cancer antigens are unknown. Here, we introduce tumor-associated circulating NK cell profiling (CNKP) as a stand-alone cancer diagnostic modality with a liquid biopsy. Metabolic profiles of NK cell activation as a result of tumor interaction are detected with a SERS functionalized OncoImmune probe platform. We show that the cancer stem cell-associated NK cell is of value in cancer diagnosis. Through machine learning, the features of NK cell activity in patient blood could identify cancer from non-cancer using 5uL of peripheral blood with 100% accuracy and localization of cancer with 93% accuracy. These results show the feasibility of minimally invasive cancer diagnostics using circulating NK cells.

## Abstract (web summary)

NK cells can be affected by tumour cells and this difference could be utilised as a cancer diagnostic. Here the authors use a nickel based plasmonic spectroscopy system to measure metabolic differences in NK cells that have been exposed to cancer cells as a method of cancer detection.

## Introduction

Natural killer cells (NK cells), a subpopulation of lymphocytes demonstrate spontaneous cytotoxicity towards tumors and viruses^1^. NK cells are part of the innate immune system with an essential function as the first line of defense against cancer development^2,3^. Moreover, NK cells do not require previous sensitization to recognize tumors^4^. As NK cells can discriminate between cancerous cells from other healthy cells, the diagnostic value of NK cell activity is significant. The field of immunotherapy has gained momentum in the last few years^5–7^, but the possibility of cancer diagnosis with NK cells has been overlooked. In adults, NK cells account for 5% to 20% of total lymphocytes in circulation^8^. As a result, NK cells will provide a realistic opportunity for cancer diagnosis. We hypothesize that the presence of tumor will reflect in the metabolic profile of NK cells as a result of NK cell activation during tumor interaction. The signals derived from such an activated state and quiescent NK cell state in circulation will enable accurate cancer diagnosis.

Cancer heterogeneity, a hallmark of cancer and cancer stem cells (CSCs) have a critical activity in tumor initiation, sustained proliferation and maintenance of tumor and help in metastasis. Cancer diagnosis research has established the importance of CSCs, a highly tumorigenic subset of tumor cells demonstrating migration and apoptosis resistance^9^. These fundamental building blocks of carcinogenesis and cancer evolution are positively correlated to therapeutic failures, drug resistance, and tumor relapse^10–12^. NK cells show preferential cytotoxicity towards CSCs as compared to the differentiated counterparts^13–15^. The susceptibility of CSCs towards NK cells is attributed to the upregulation of cytotoxicity receptors on NK cells and their respective ligands of CSCs. Moreover, CSCs enriched after antiproliferative therapies show increased expression of stress ligands resulting in NK cell sensitization^16^. Therefore, targeting the signals of CSC-associated NK cells may prove to be an effective cancer diagnostic approach.

For detection of the rare signals of tumor-associated NK cells in circulation, an ultrasensitive sensor is necessary. It is very difficult to identify tumor-associated NK cells as most cancer antigens are unknown. Currently, there is no diagnostic tool to detect tumor-associated NK cells in circulation. Therefore, a marker-free approach was necessary. Furthermore, the use of blood samples without cellular isolation was essential to preserve the integrity of the rare signals of NK cell interaction with tumor^17^. The ultrasensitive technique of Surface-enhanced Raman scattering (SERS) was adopted as the detection methodology. SERS has demonstrated the capability of marker-free diagnosis^18^. SERS interconnects biomolecular chemistry and physics to biomolecular functions. Exploration of the complex NK Cell functional signatures can be achieved intuitively with SERS^19^. However, SERS nanostructures are well known for significant challenges in achieving reproducible and uniform Raman response. One-dimensional and two-dimensional distribution of hot spots suffers from limited density resulting in inhomogeneous signal distribution and non-reproducible signals. Uniform SERS active sites are extremely difficult, expensive, and time-consuming to produce. To overcome this limitation, we developed a nickel-based metal-semiconductor probe system for the generation of stable and reproducible signals. Nickel is an inexpensive catalyst with numerous critical features, including oxidative addition and easy access to different oxidation states, which are significant in the development of metal-semiconductor nano systems^20–22^. These fundamental properties of nickel enable a wide range of applications including SERS. The plasmonic enhancement of nickel is widely reported^23–29^ in addition to the charge transfer-based enhancement. Consequently, the introduction of a hybrid material composed of nickel and nickel oxide results in not only a significantly improved SERS signal but also a signal that can be reproduced, which is extremely valuable for cellular diagnostic applications with semiconductor-based nanosensor technologies. Such materials (metal-semiconductor) will also make it possible for more substances to be candidates for Raman sensing. Synthesis of large quantities of nanomaterial is equally challenging. Here we have demonstrated the use of femtosecond laser fabrication methodology, which is amendable for mass production useful in large-scale research on cellular structures and intracellular signals. Additionally, for the sensor to be robust, highly reproducible signals are required. The use of the multiphoton ionization mechanism of ultrashort femtosecond pulsed laser results in uniform production of multi-mode probes that are highly reproducible. The modern fiber-oscillator/ fiber-amplifier design of this laser provides high spatial mode quality with very low noise performance. The fiber amplifier of this laser minimizes variation of energy levels. This results in very stable peak power and ionization energy performance resulting in the synthesis of nanoparticles with minimal distribution and dimension variation. “OncoImmune probe platform” was synthesized with 3D networks of nickel nanoprobes. The shape of the probes was tuned for ultrasensitive detection.

In this work, we explore the feasibility of using the signals derived from cancer-cell-associated NK cells as well as CSC-associated NK cells as a stand-alone cancer diagnosis methodology. OncoImmune probe platform functionalized with SERS capability is introduced for undertaking circulating NK cell profiling (CNKP) for cancer diagnosis. This is achieved by atomic scale narrowing of the probe apices resulting in a substantial increase in the localized surface plasmon resonance (~650-fold enhancement with a limit of detection of up to femtomolar (10^−15^ M) concentration). This marker-free approach enables the generation of the holistic profiles of the metabolic states of NK cells. SERS profiling of NK cells associated with hard-to-detect cancers (triple negative breast cancer, small cell lung cancer, colorectal adenocarcinoma cancer encompassing about 60% of cancer cases) is achieved with single-cell sensitivity with highly reproducible signals. CNKP of cancer-cell-associated NK cells and CSC-associated NK cells demonstrates well-defined, distinct, cancer-specific signals. We experimentally show the preferential targeting of CSCs by NK cells compared to cancer cells with a 1.4-fold increase (*p* < 0.0001 *t* test) in activation for CSC-associated NK cells. This also demonstrates that the use of CNKP of CSC-associated NK cells is useful for cancer diagnosis. The machine learning model trained with SERS signals of NK cell activity in cell culture can identify cancer from non-cancer with a very small amount of peripheral blood (5 µL) without the need for cellular isolation with 100% prediction accuracy. Localization of tumor shows a prediction accuracy of up to 93%. As the training data is obtained from easy to collect cell-culture, this approach eliminates the disadvantages of insufficient human data for training. By utilizing tumor-associated NK cell signals in peripheral blood, CNKP has the potential to improve minimally invasive cancer diagnostics.

## Results and discussion

### Prediction of tumor-associated NK cells for cancer diagnosis method

**Fig. 1:**
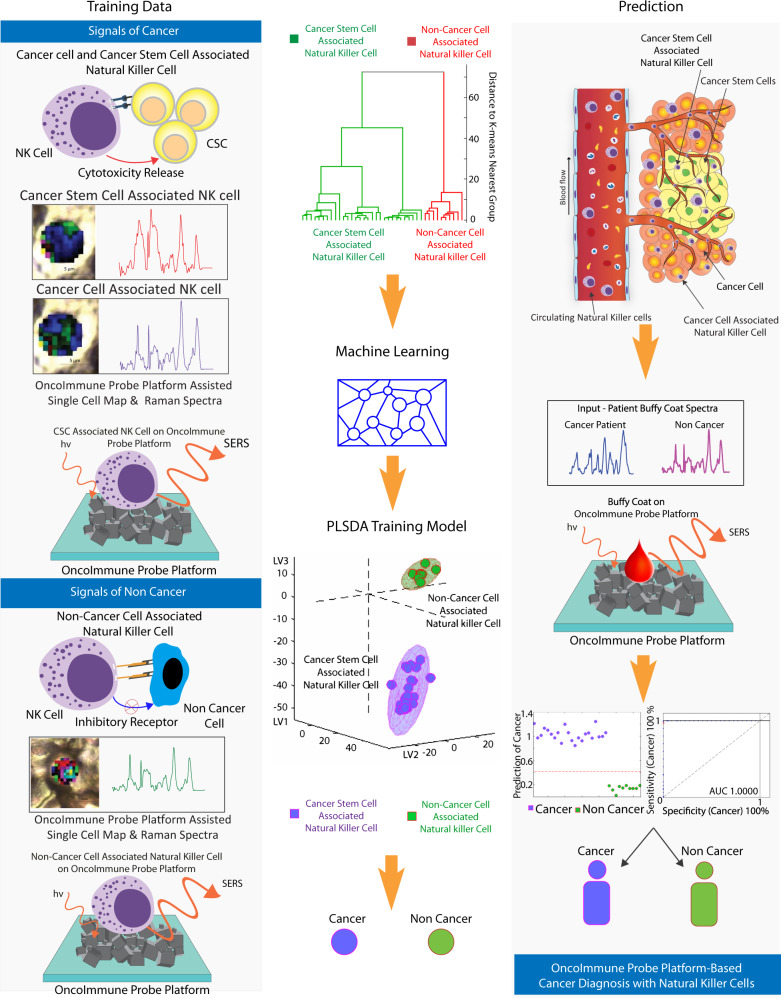
Schematic representation of working of circulating natural killer (NK) cell profiling (CKNP) with OncoImmune probe platform. Left panel demonstrates training dataset collection with tumor (cancer—purple spectra and CSC-associated NK cell—red spectra) and non-cancer-cell-associated NK cell Raman profile—green spectra. Middle panel demonstrates model learning. Exploratory analysis with K-means clustering was performed. PLSDA (Partial Least Squares Discriminant Analysis) was then applied. Right panel depicts schematic of circulating NK cells interacting with cancer and cancer stem cells. A small volume (5 µl) of buffy coat (cancer patient—blue spectra non-cancer—pink spectra) was dropped on the OncoImmune probe platform and Raman spectra were obtained. Analysis of the spectra based on the similarity of NK cell activity using machine learning algorithm demonstrated very high accuracy.

In this study, we report that molecular probing of NK cells has the potential to provide diagnostic information for cancer patients. As CSCs are resistant to antiproliferative therapies and have the ability to repopulate bulk tumor^30^, it is important to identify CSCs. In this study, the existence of CSCs was determined by observing changes in NK cell expressions. To detect the presence of CSCs, NK cells were selected for several reasons. NK cells forming the critical part of the innate immune system, are the first line of defense against cancer and are responsible for the cancer immune surveillance^3^. Additionally, NK cells do not require any prior sensitization to recognize tumors^4^. Moreover, amongst all immune cells, only NK cells demonstrate preferential cytotoxicity towards CSCs^16,31,32^. Although CSCs are able to escape other immune cells, CSCs cannot escape NK cell surveillance and demonstrate vulnerability towards NK cells. Therefore, we hypothesize that the presence of CSCs will naturally activate NK cells with signature molecular changes, enabling identification of CSCs and hence the presence of cancer. Figure 1 illustrates this diagnostic approach. For this purpose, NK cells were cocultured with cancer cells as well as CSCs. This led to NK cells exhibiting three phenotypes based on cell-specific association. Consistent with this idea, we obtained naïve NK cell spectra, cancer-associated NK cell spectra, and CSC-associated NK cell spectra from cell culture. The three phenotypes form the basis for the distinction of cancer diagnosis in this study. Analysis of SERS spectra of human blood samples based on the similarity to the SERS spectra of NK cell activity using a simple machine learning algorithm was undertaken. We hypothesize that the Raman signals of NK cell interaction with cancer cells and CSCs can be detected from patient blood. Thus, we first cocultured NK cells with cancer cells, CSCs and non-cancer cells and collected SERS signals using SERS functionalized OncoImmune Probe Platform.

In this study, machine learning (ML) - a subfield of artificial intelligence that has evolved rapidly in recent years was adopted for prediction. Unlike conventional techniques, ML techniques have the capabilities of addressing complex problems involving massive combinatorial spaces or nonlinear processes without incurring massive computational costs^33^. We have explored the use of ML by adopting the ML approach for cancer diagnosis, to address the complex molecular fingerprinting of tumor-associated NK cells for prediction of cancer. ML tools have consistently generated, tested, and refined scientific models^34,35^. This family of statistics-based methods that can make predictions of properties of molecules and materials without invoking computationally demanding electronic structure calculations has the potential to accelerate a variety of applications in chemical and molecular sciences including Raman spectroscopy. The spectral dataset from co-culture was used to train the machine learning model with binary classification (cancer & non-cancer). The supervised model successfully classified the co-culture data into two clusters. Human blood samples of cancer and non-cancer were also classified through this model.

### Synthesis characterization of OncoImmune probe platform for in-silico detection of cancer

**Fig. 2:**
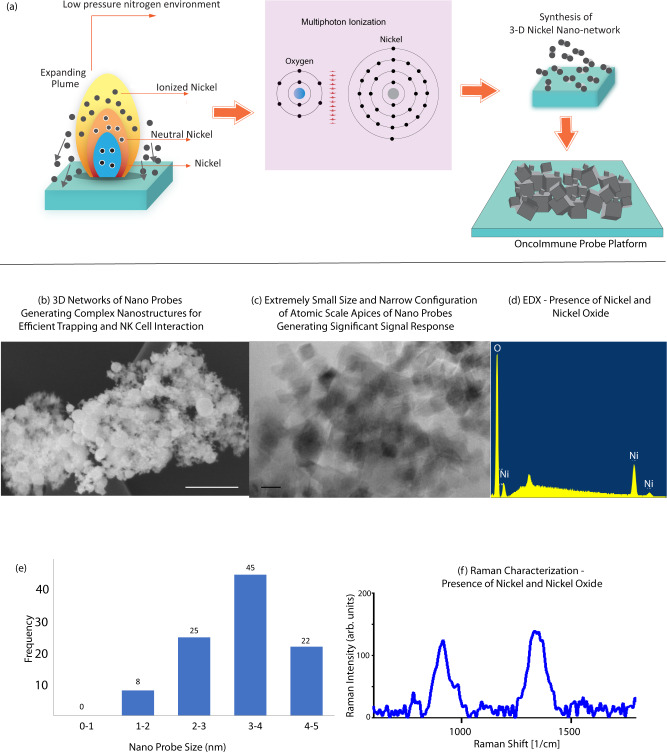
Synthesis and characterization of OncoImmune probe platform for in-silico detection of cancer. **a** Schematic representation of multiphoton ionization resulting in the formation of intricate 3D nickel nano network of the Oncoimmune probe platform. **b** High-resolution scanning electron microscopy (HRSEM) image of the OncoImmune probe platform demonstrating 3D networks of the probes generating maze-like sensing platform. Scale bar = 200 nm. **c** High-resolution transmission electron microscopy (HRTEM) demonstrating the small size of the probes. Scale bar = 5 nm (**d**) energy dispersive x-ray spectroscopy (EDX) showed the presence of nickel and nickel oxide-based probes in the Oncoimmune platform. **e** Probe size distribution frequency. Samples *n* = 100 independent particles. For particle size calculation, 100 independent particles were measured, and five independent experiments were performed. **f** Raman spectra demonstrating the presence of nickel and nickel oxide peaks. Raman measurements were taken 10 times and averaged.

We successfully synthesized cubiform networks of nickel nanoprobes using an ultrafast femtosecond laser. Figure 2a shows a schematic of femtosecond laser ablation on nickel substrate generating nickel ions, neutral species, and nickel radicals. The Yb-doped ultrafast, femtosecond laser pulse consists of high-energy photons that arrive in a near-simultaneous (10^−15 ^seconds) interval^36^. High peak power facilitated the augmented ablation of the nickel substrate as more than one photon can interact simultaneously facilitating direct multiphoton ionization (MPI). MPI of the nickel substrate caused excitation of the electrons from the valence band to the conduction band. These free electrons acquired more energy by inverse bremsstrahlung (IB) absorption, wherein the electrons acquire more kinetic energy from the incoming photon^37^. MPI together with IB absorption created an electron avalanche causing further ionization. As the duration of the pulse is much shorter than the time taken by nickel electrons to conduct heat, there is a phase change where the solid nickel becomes a super-heated liquid. Above, the ablation threshold strong atomization and ionization of the super-heated liquid occurs and results in explosive boiling of the liquid with subsequent vaporization. This phase change caused an irreversible breakdown of the nickel crystalline structure into its constituent nickel ions, molecules, neutrals, and electrons. The kinetic energy of the ejected ions is considerably high, and the peak kinetic energy of nickel ions is estimated to be around 0.5 KeV^38^. Above and around the ablation area, an evaporation layer known as the Knudsen layer is formed, which contains ions, atoms, and molecules^39^. With continuous vaporization, the plume gets supersaturated, and nucleation begins to appear. The presence of background gas altered the expansion kinetics of the plume and resulted in a change in the shape and chemical composition of the nanoparticles. Under low-pressure nitrogen, two observations were made. First, nitrogen gas increased the condensation of vapor and resulted in the formation of smaller nanoparticles, as there is less time for nuclear growth. Second, the faster condensation and the supersaturated plasma increased the pressure within the plume and resulted in cubic-shaped nanoparticles with sharp edges are formed. Under low-pressure oxygen gas, the trend was reverse with multi-faceted roughly spherical nano shapes being produced with dimensions bigger compared to nanoparticles made with low-pressure nitrogen gas^40^. Figure 2b shows scanning electron microscopy (SEM) image of synthesized nanoparticles. Surface morphology and topography indicate a network of nano-scale probes in three-dimensional layered assemblies. Figure 2c shows a transmission electron microscopy (TEM) image of the probes showing the small size of probe with sharp features (Supplementary Figs. 1a–c). The small size favors excellent SERS signal. Particle size analysis was done using ImageJ software. The median particle size was 4.3 nm (Supplementary Fig. 1d).

Energy dispersive x-ray spectroscopy (EDX) was done to understand the chemical composition of the probe containing nickel metal components together with nickel oxide components. Figure 2d shows the majority of nickel peaks and an oxygen peak. Figure 2f shows Raman characterization of OncoImmune probe sensor with nickel and nickel oxide peaks. X-ray photoelectron spectroscopy (XPS) was undertaken for analysis of surface functional groups. Supplementary Figure 2 shows the XPS- *O1S*, *C1S,* and *Ni2P* spectra which provided information on the material composition as well as the surface properties of the probes. The peak positions are presented in Supplementary Table 1. On curve fitting of *Ni2P* spectra with Gaussian function, the presence of two spin-orbital regions representing *Ni 2 p1/2* at the binding energy of 856 eV and *Ni 2p 3/2* at the binding energy of 862 eV were evident^41^. Shoulder shake-up peaks at higher binding energies 874 eV and 880 eV were also present. The energy difference of 17 eV between the *Ni 2p3/2* and *Ni 2p1/2* supports the existence of nickel oxide (NiO)^42,43^. The curve fitting of *O1S* spectra showed four distinct peaks. The peak at 529 eV was assigned to core level of O^2-^ anions. The peak at 531 eV was assigned to the lattice Oxygen and the peak at 533 eV was assigned to the defective sites within the oxide crystal, adsorbed oxygen or hydroxide groups while the small peak at 534 was due to the adsorbed water^44^. The *C1S* spectra showed peaks at 284 eV assigned to C-C and C–H hydrocarbon states. The peak at 286 eV was due to C–OH and C–O bonds while the peak at 288 eV was attributed to carbon atoms bound to oxygen with double bond C = O^43^.

### Molecular level detection with OncoImmune probe platform

**Fig. 3:**
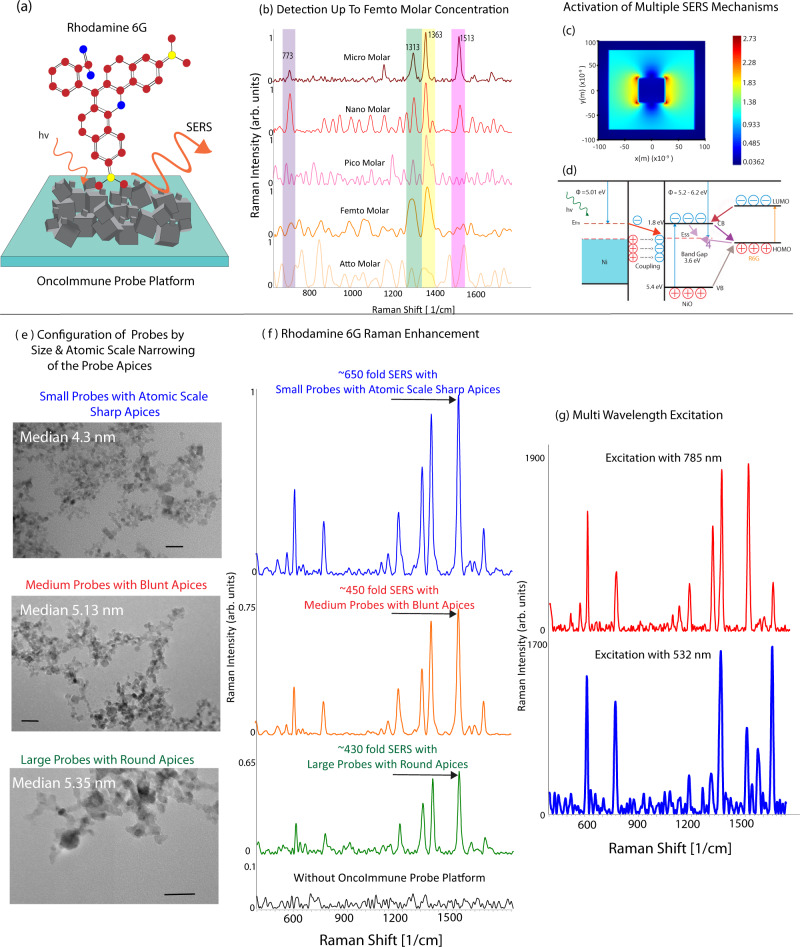
Molecular level detection with OncoImmune probe platform. **a** Schematic representation of Rhodamine 6G analyte on OncoImmune probe platform. **b** Detection of analyte at femtomolar concentration was achieved with nanoprobes with sharp apices (representative spectra at micromolar—maroon, nanomolar—red, picomolar—pink, femtomolar—orange, attomolar—yellow. Demonstration of multiple SERS mechanisms with (**c**) Finite-Difference-Time-Domain (FDTD) simulation of Ni probes demonstrating the presence of localized surface plasmon resonance and (**d**) schematic representation of charge transfer mechanism. **e** TEM images of sharp & cubical, blunt, and round probes with median diameter of 4.3 nm, 5.13, and 5.35, respectively. scale bar = 5 nm. **f** SERS enhancement with R6G observed with sharp, blunt, and round probes were 650-fold (blue), 450-fold (orange) and 430-fold(green) enhancement respectively. **g** Wavelength dependent SERS at 532(blue) and 785(red) nm demonstrates presence of charge transfer. All spectra shown are an average of five spectra. Source data are provided as Source Data File.

To detect a particular population of NK cells associated with a tumor, we require an ultrasensitive sensor capable of detecting minute concentrations in blood. Therefore, we evaluated the sensitivity of the probe using the hematologically compatible leukocyte marker R6G (rhodamine 6 G) (Fig. 3a). As shown in Fig. 3b, the peaks at 612 cm^−1^, 773 cm^−1^, 1126 cm^−1^, 1183 cm^−1^, 1313 cm^−1^, 1363 cm^−1^, 1513 cm^−1^ and 1651 cm^−1^ were evident. The detailed Raman assignment is presented in Supplementary Table 2. We tested three probes to optimize the sensor. Figure 3e shows TEM images of the three types of probes. We observed highest SERS ~ 650-fold with the probes with the smallest size (median size 4.3 nm) and sharp cubical geometries. The medium-sized probes (median size 5.13 nm) with partially rounded corners demonstrated ~450-fold enhancement. The probes with the largest size (median size 5.35 nm) with round geometries demonstrated lowest enhancement ~430-fold enhancement. Although the size of the probes was instrumental in increasing the surface area, the probes had a very similar particle size distribution. Therefore, we hypothesize that the substantial variation in the enhancement was due to the change in the geometry (Supplementary Fig. 3a, b, e, f)).

As shown in Fig. 3b, the limit of detection of R6G was done with varying concentrations of R6G and their corresponding SERS signal. We used 1 × 10^−6^, 1 × 10^−9^, 1 × 10^−12^, 1 × 10^−15^ and 1 × 10^−18^ M concentrations of R6G. The SERS intensity was observed for all the peaks except at attomolar concentrations. We were able to detect the Raman signal of the analyte in femtomolar range which is necessary for the rapid detection of trace levels of tumor-associated NK cells. The SERS enhancement was substantially increased a combination of different mechanisms that bring about intense SERS enhancement in the synthesized nickel probes (Fig. 3c, d). The interconnected nickel nanoprobes geometries were capable of augmenting the electromagnetic field through collective free electron oscillations^45^, at resonant frequency called localized surface plasmon (LSPR)^46,47^. Maxwell’s equation for electrical field enhancement was solved for a defined boundary condition using FDTD simulation^48,49^. The finite element simulation model was created with ANSYS software to assess the electrical field strength in the proximity of nickel nanoprobes. The result obtained for 5 nm nanoprobes in dielectric medium on irradiation with 532 nm excitation source and 785 nm excitation source is presented in Supplementary Fig. 3a, b. The simulated data shows the average electrical field enhancement on the surface to be 0.485 eV and 0.461 eV for 532 and 785 nm excitation, respectively. The electrical field enhancement at a single hotspot (sharp edges of the cubes) were calculated to be 2.73 eV and 2.62 eV at 532 and 785 nm respectively (Supplementary Fig. 3a, b). The intensity of enhancement is directly proportional to the fourth power of the amplitude of the electric field (*E*_elec_) in the region of the probes (*E*_scat_ α *E*_elec_^4^). Additionally, when the number of probes was increased, the electrical field enhancement also increased substantially (1.4-fold with two probes). Therefore, the presence of hundreds of small probes in the laser spot will exponentially improve the signal. This in addition to the presence of multiplicative charge transfer resonance has resulted in substantially enhanced SERS.

The large surface area of the probe surface allows the adsorption of molecules on the surface of the probe. R6G was used as analyte to investigate SERS activities of nickel nanostructures due to their excellent adsorption on the nickel surface^50^. The adsorbed molecule in contact with nickel nanoprobes displays charge transfer effect due to Herzberg-Teller vibrionic coupling. The wavelength-dependent SERS shows selective enhancement of b2 modes of R6G molecule (Fig. 3d). The Herzberg–Teller vibrionic coupling is brought by the interaction of excited state (LUMO) of R6G molecule and fermi level electrons in the nickel^51^. The net result of this interaction is a broadening of the virtual level of the molecule and charge transfer between metal and R6G molecule. As shown in supplementary Figure 3g, charge transfer is also evident from the UV–Vis absorption spectra of R6G molecule with nickel nanoprobes compared to pure nickel probes and pure R6G. In addition to the plasmonic and charge transfer mechanism, nickel metal enhances the electromagnetic field of NiO by forming a strong local electric field at Ni/NiO interface. Since the work function of Ni (5.01 eV) is much smaller than NiO (5.2-6.2 eV), the electrons are transferred from nickel to NiO, increasing the negative charge in the interface close to NiO and equal positive charge in the interface close to nickel. Metallic Ni influence the near band-edge emission of NiO and electrons from the interface move to conduction band. This increased local electrical field facilitates SERS^52^. Additionally, the nickel nanoprobes system has materials with various refractive indices (nickel is ~1.96, NiO is ~2.8 and air = 1)^53^. These varying refractive indices allow multiple light scattering^54^ and multiplication of Raman photons thereby improving Raman enhancement^55^.

Additionally, the 3D interconnected networks have a critical function in the overall SERS amplification. Multiple SERS active sites were introduced due to the presence of 3D networks resulting in localized surface plasmon resonance at the complex formation^56^. SERS intensity can be determined by the width of individual interparticle gaps^57^. In this case, the presence of narrow width gaps as well as larger width gaps is evident in the 3D structures of probes as shown in the TEM. FDTD simulation as per Supplementary Fig. 3c, d demonstrated substantial increase in the intensity of LSPR with greater number of hot spots. The multiple layers of the 3D nanostructures had dual function—first to produce plasmonic enhancement and second to adsorb analyte molecules resulting effective charge transfer. The multiplicative effect of the plasmonic enhancement due to the localization of light in the multiple layers of ultra-small gaps in the probes and charge transfer enhancement resulted in substantially enhanced signal response^58^.

### OncoImmune probe platform assisted trapping and circulating natural killer cell profiling for cancer diagnosis-CNKP

**Fig. 4:**
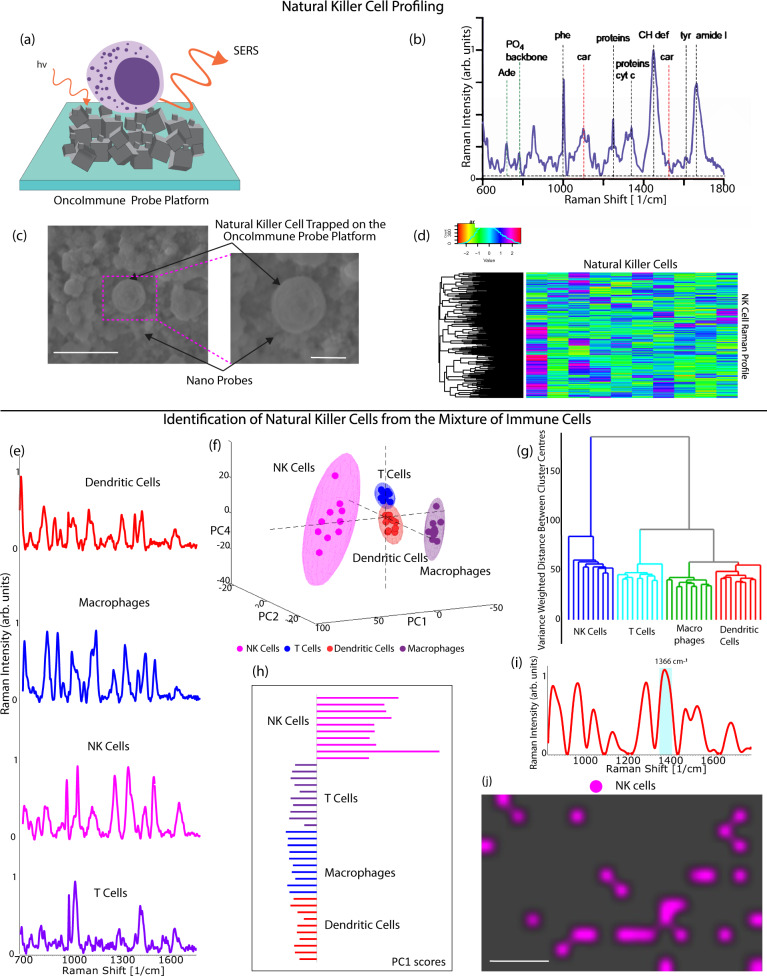
Immune cell profiling for cancer diagnosis. Circulating natural killer cell profiling (CNKP) using SERS (**a**) Schematic representation of NK Cell on OncoImmune probe platform (**b**) Representative Raman spectra of NK cell demonstrating presence of various biomolecules of NK cells (**c**) SEM image of NK cell trapped on the OncoImmune probe platform. Scale bar = 3 um and 1um respectively (**d**) Heatmap of the holistic Raman profile of NK cells demonstrating correlated cellular biomolecules. Identification of NK cells from the mixed population of PBMCs (**e**) Representative Raman spectra of various types of PBMCs—dendritic cells (red), macrophages(blue), NK cells(pink) and T cells(purple). Source data are provided as Source Data File. **f** Principal component analysis demonstrating clear clusters differentiating the immune cell types (**g**) Hierarchical cluster analysis support PCA analysis demonstrating unique fingerprints of PBMCs (**h**) Score plot of PC1 demonstrating variation in the signals of NK cells (**i**) PC1 loading to define signature peaks of NK cells for distinction in the mixed population of PBMCs (**j**) NK cell mapping of the OncoImmune probe platform. Scale bar = 20 μm. Raman measurements were taken 10 times and averaged.

Human peripheral blood mononuclear cells are composed of a diverse array of immune cells, including T cells, NK cells, Dendritic cells, and monocytes. We began by demonstrating the OncoImmune probe platform capacity to discriminate between various types of PBMCs. As seen in Fig. 4e, the spectra of diverse cell types exhibit unique characteristic peaks. The spectra are distinguishable because of the distinct Raman shifts observed for different types of cells. The principal component analysis demonstrated clear clustering of cells into four separate groups (Fig. 4f). Additionally, the hierarchical cluster analysis showed a dendrogram with distinct clustering (Fig. 4g). As a result, we determined that NK cells may be recognized from other types of immune cells by their distinct characteristic spectra. PCA (Principal Component Analysis) was used to characterize NK cells. PCA decreases the dimensionality of data by decomposing the Raman spectra mathematically into primary components. The first principal component showed a positive association between NK cells and all other cells and a negative correlation between all other cells (Fig. 4h). Using this feature, we defined the NK cell signature’s strongest peak in the PC1 loading spectra (Fig. 4i). Then, PBMCs were mapped using Raman spectroscopy. As seen in Fig. 4j, we were able to successfully demonstrate the presence of NK cells in the PBMC mixture. This investigation established unequivocally that circulating NK cells are capable of generating distinct NK cell signature spectra that are highly distinguishable from all other PBMC spectra and may be utilized as a cancer detection marker.

Next, we investigated the metabolic states of NK cells using NK-92 cell line and primary NK cells. Figure 4b shows signature Raman spectra of NK-92 cell with characteristic bands from carbohydrates, proteins and lipids. Raman assignment of all biological peaks reported in this study are tabulated in Supplementary Table 3. Noticeable peaks are phenylalanine band at 1003 cm^−1^, CH deformation at 1450 cm^−1^ and amide I at 1661 cm^−1^. However, the most distinct bands typical of lymphocytes are seen at 1522 cm^−1^ and 1158 cm^−1^ belonging to carotenoids^59^. Carotenoids are robust Raman scatterers and have characteristic Raman spectra. The presence of carotenoids in NK cells is an indication of cytotoxicity and cell surface activation. Other spectral peaks originate from enzymes in the dense granules within the cytoplasm^60^. Figure 4d demonstrates heatmap of the holistic Raman profile of NK cells demonstrating correlated cellular biomolecules. Supplementary Figure 4a show the primary NK cell spectra. The Raman shifts of all major peaks of primary NK cell were identical to NK-92. Additionally, PCA was used to demonstrate the similarity between NK92 and primary NK cells. As can be observed from the PCA, the first three major components were merged into a single cluster for NK92 and primary NK cells, demonstrating the two cell types’ strong similarities (Supplementary Fig. 4b).

NK cell function is dependent on the balance between inhibitory and stimulatory signals received through interaction with tumor cells. When the stimulatory signals are more than the inhibitory signals, NK cells mediate the killing of tumor cells. Hence, NK cell cytotoxicity is affected by the dynamic environment the cell interacts with. In patients with cancer, the number of NK cells is increased. However, quantification of NK cells is often a poor indicator of the cause and severity of the disease. Therefore, we wanted to analyze the quality of NK cell by investigating the metabolic state of NK cell to see the changes in the tumor microenvironment. In this study, we hypothesize that NK cells undergo metabolic changes on interaction with cancer cells and CSCs. Our findings support our hypothesis that NK cells faithfully reproduce the changes in tumor cellular environment. We find that NK cells change from inactive to active and vice-versa upon interaction with cancer cells and CSCs. The holistic analysis of molecular properties of the cells was possible due to the efficient probe-cell interaction. First, the 3D interconnected structures of the OncoImmune probe platform entrapped the cells (Fig. 4c). The surface functionalization permitted cellular contact with the nanoprobes. As previously shown in the XPS analysis, the presence of oxygen containing groups such as –OH, C–O, C=O, C–OH incorporated on the surface of the nanoprobes, instrumental in efficient probe-cell interaction^61–63^. Substantially enhanced signal response was attributed to the efficient probe-cell interaction.

### Ultrasensitive detection with OncoImmune probe platform assisted CNKP

**Fig. 5:**
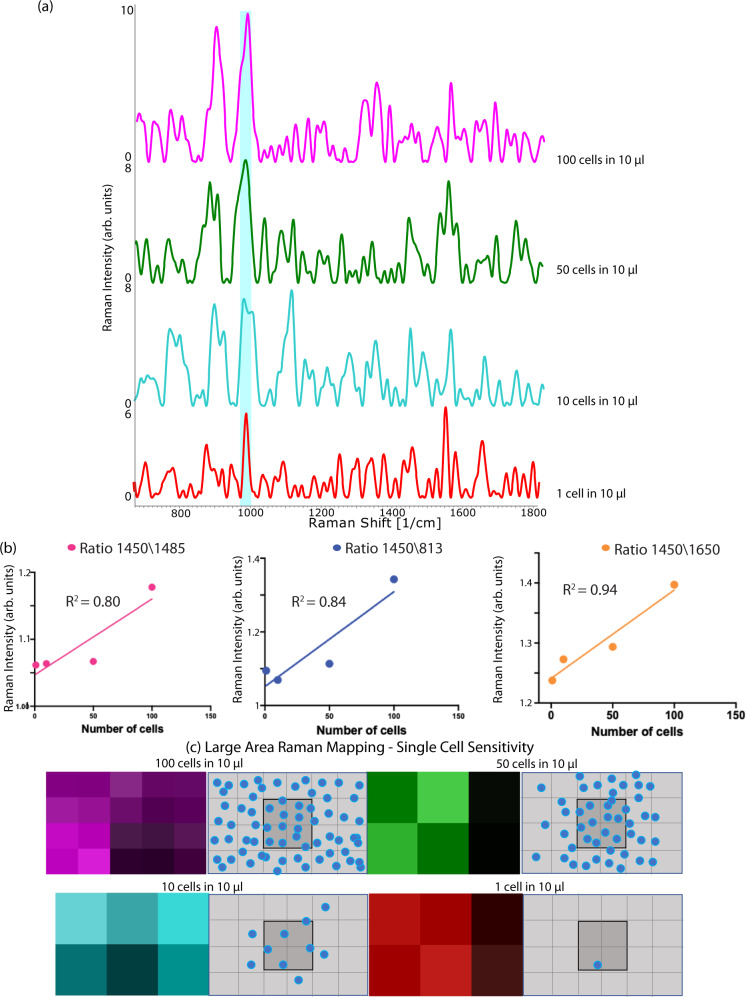
Single-cell sensitivity of OncoImmune probe platform. **a** Raman spectra of cells at 100 cells per µl (purple), 50 cells per µl (green), 10 cells per µl (cyan) to 1 cell per µl (red) cell density. Source data are provided as Source Data File. **b** Ratio analysis at I1450/I1650 (lipid/protein)—pink line, I1450/I813 (lipid/RNA)—blue line and I1450/I1485 (lipid/DNA)—orange line demonstrated increasing trend with increasing concentration (R2 0.94, 0.84, 0.80 respectively). **c** Large area mapping—100 cells per µl (purple map), 50 cells per µl (green map), 10 cells per µl (cyan map) to 1 cell per µl (red map) of OncoImmune probe platform demonstrating the ability to recognize NK cell signals at single-cell level. The gray maps with blue dots are schematic representation of cells on OncoImmune probe platform. Raman measurements were taken 10 times and averaged.

NK cells have been known for their ability to selectively recognize and kill CSCs. However, in circulation, the amount of CSC associated with NK cell is very less. Therefore, we need ultrasensitive sensor that can detect trace levels of tumor-educated NK cells in circulation. Surface-enhanced Raman scattering (SERS) was adopted for ultrasensitive detection. SERS-based methods, very useful for monitoring intracellular proteins and other macromolecules such as lipids and nucleic acids enabled intensive analysis of cellular biochemical composition^64^. SERS-based molecular fingerprints at the sub-cellular level will allow real-time information in a non-destructive way^65^. This marker-free approach will be useful for the analysis of minute intracellular changes^66^. Variation in the biomolecular expression can be captured by the OncoImmune probe platform very effectively. The ability of the sensor to detect the NK cells even at a single-cell level will enable the detection of rare CSC-associated NK cell signals in circulation. To show single-cell sensitivity of Oncoimmune probe platform Raman spectra of NK cells with serial dilution (from 100 NK cells in 10 µl to 1 NK cell in 10 µL) was undertaken (Fig. 5a). As shown in Fig. 5b, the increase in Raman intensity was directly proportional to the number of cells captured on the sensor. SERS intensity ratio *I*_1450_/*I*_1485_ (lipid/DNA), *I*_1450_/*I*_813_ (lipid/RNA), and *I*_1450_/*I*_1650_ (lipid/protein) demonstrated increasing trend with increasing concentration for immune cell capture across all spectra of different concentrations. The linear regression for the number of immune cells captured by the sensor to the Raman intensity is shown in the figure from 100 cells to a single cell (*R*^2^ 0.80, 0.84 and 0.94 for *I*_1450_/*I*_1485_, *I*_1450_/*I*_813_, *I*_1450_/*I*_1650_ respectively). Figure 5c shows the high-resolution single-cell Raman mapping of NK cells demonstrating ability to detect cells at single-cell level. Supplementary Figure 5a shows TEM images of the platform taken at different sites and a fair distribution of nanoparticles can be seen. An average of 15 particles can be seen per random site (*n* = 4, S.D 2.5) (supplementary figure 5b). Additionally, to show the excellent reproducibility, the relative standard deviation (RSD) was calculated using two SERS analytes (crystal violet, methyl orange) and patient buffy sample. By selecting 9 random spots, the RSD was calculated with 1176 cm^−1^, 1111 cm^−1^ and 1000 cm^−1^ as the reference peak for crystal violet, methyl orange and buffy sample, respectively. Supplementary Figure 6 shows the uniform signal intensity with an RSD of 3.25 (crystal violet), 3.92(methyl orange) and 10.98% (buffy). The high reproducibility resulted in very low relative standard deviation demonstrating the robustness of this approach.

### Applicability of cancer-associated NK cells for cancer diagnosis

We collected SERS spectral profile data from three cancer-associated NK-92 cells (breast carcinoma (MDA-MB231), small cell lung carcinoma (H69-AR) and Colorectal cancer (Colo 205) by co-culturing NK92 with cancer cells for 24 h. After 24 h, 5 µl of the cocultured and tumor-associated NK-92 cells were dropped on the OncoImmune platform and SERS spectral profile was obtained using 785 nm wavelength. Control NK-92 cells were grown separately, and SERS spectra were obtained at 785 nm for comparison. The control NK-92 spectral profile for non-cancer-associated NK cells correlated with the previously reported SERS spectral profiles of NK cells^67,68^. Casual observation of the SERS spectra of tumor-associated NK cells and non-tumor-associated NK cells show unexpected differences between control and tumor-associated NK cell spectra and between the different cancer-associated NK cells spectra.

NK cells that are completely devoid of any tumor cell association showed characteristic Raman spectra with characteristic bands from carbohydrates, proteins, and lipids. This profile can be correlated to the NK cells in circulation. Figure 4 (b) show the signature Raman spectra of circulating NK cell. The band at CH deformation at 1450 cm^−1^ and amide I at 1661 cm^−1^, amide II band at 1555 cm^−1^ and amide III band at 1337 cm^−1^ can be seen. Other spectral features are disulfide bonds (S–S) between 500 and 550 cm^−1^ and aromatic amino acids at 1004 cm^−1^ (from phenylalanine), 830 cm^−1^ and 854 cm^−1^ (from tyrosine doublets), 1340 cm^−1^ of tryptophan^69^.

**Fig. 6:**
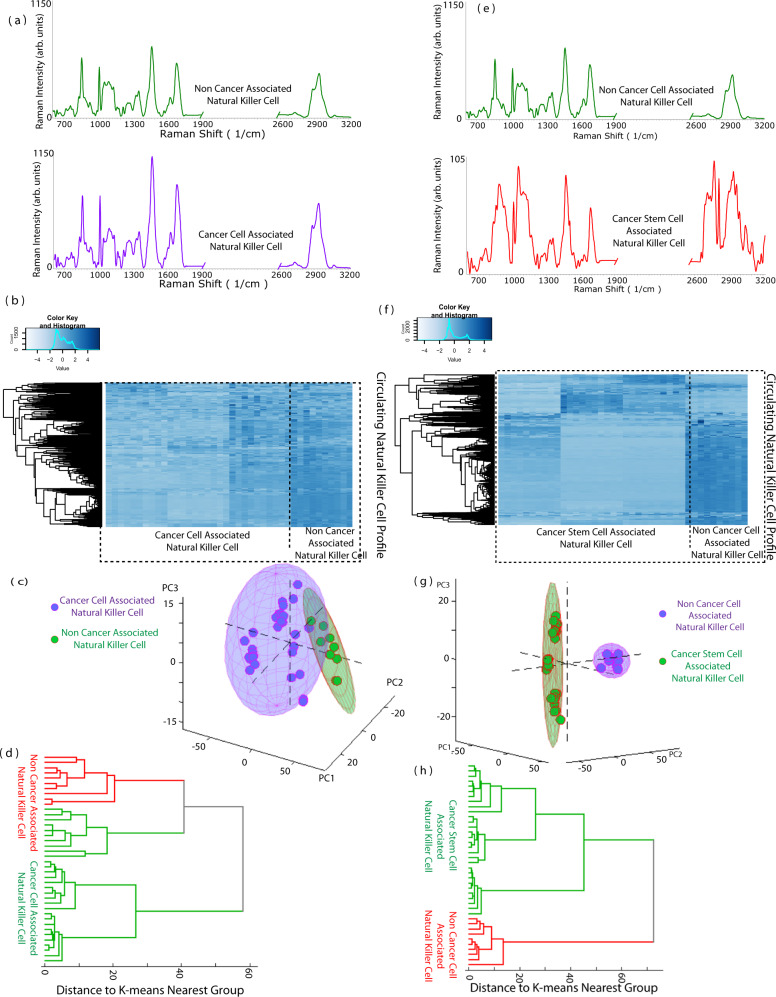
Variation in the signals of cancer-associated NK cell, CSC-associated NK cell and non-cancer-associated NK cell. **a** Representative Raman spectra of non-cancer-cell-associated NK cells (green) and cancer-cell-associated NK cells (purple). **b** Heatmap of cancer-associated NK cell and non-cancer-associated NK cell. **c** Principal component analysis shows overlap between the clusters. **d** Dendrogram of the distance for K-Means nearest group demonstrated mixed NK Cell association signals for cancer and non-cancer. **e** Representative Raman spectra of non-cancer-associated NK cell (green) and CSC-associated NK cell(red). **f** Heatmap of CSC-associated NK cell and non-cancer-associated NK cell. **g** Principal component analysis shows clear clustering. **h** This observation is supported by dendrogram. Source data are provided as Source Data File. Raman measurements were taken 10 times and averaged.

**Fig. 7:**
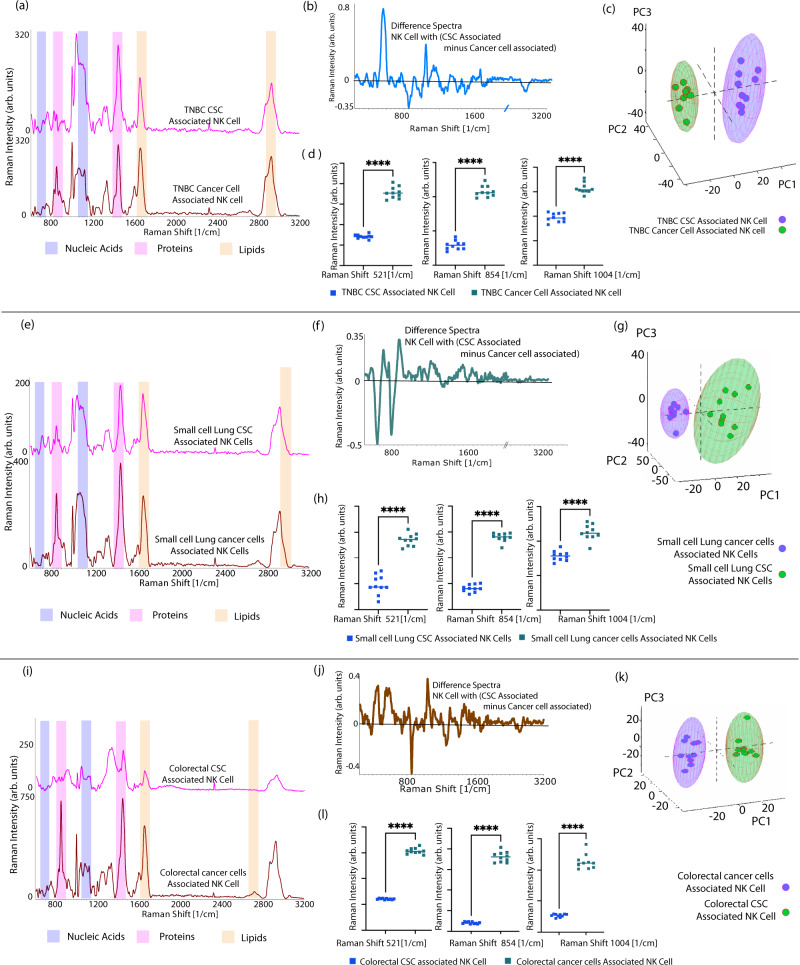
Variation in the signals of cancer cell and CSC-associated NK Cell. **a** Spectral variation for triple negative breast adenocarcinoma. Pink spectra for CSC-associated NK cell, maroon spectra of cancer-cell-associated NK cell. **b** Difference spectra for breast. **c** Principal component analysis showing clear separation of cancer-associated, and CSC-associated NK cells. **d** Two tailed *t* test of distinct peaks in difference spectra demonstrate significant variation. (****mean *P* ≤ 0.0001). **e** Spectral variation for small cell lung adenocarcinoma. Pink spectra for CSC-associated NK cell, maroon spectra of cancer-cell-associated NK cell. **f** Difference spectra for lung. **g** Principal component analysis showing clear separation of cancer-associated, and CSC-associated NK cells. **h** Two tailed *t* test of distinct peaks in difference spectra demonstrate significant variation. (****mean *P* ≤ 0.0001). **i** Spectral variation for colorectal cancer. Pink spectra for CSC-associated NK cell, maroon spectra of cancer-cell-associated NK cell. **j** Difference spectra for colorectal. **k** Principal component analysis showing clear separation of cancer-associated, and CSC-associated NK cells. **l** two tailed t test of distinct peaks in difference spectra demonstrate significant variation (****mean *P* ≤ 0.0001 *n* = 60 independent cell spectra. Source data are provided as Source Data File. Raman measurements were taken 10 times and averaged.

The spectral intensity of tumor-associated NK cells was slightly altered compared to NK cells which have not associated with tumor cells (Fig. 6a). A heatmap of circulating NK cell profiles indicates a small difference in metabolic characteristics between NK cells associated with cancer and those not associated with cancer (Fig. 6b). This can be due to NK cell recognition of tumor and switching to an active mode. Breast, lung, and colorectal cancers differ in their prognosis and treatment outcome due to their different signaling mechanisms and molecular pathways. Consequently, the immune responses vary between different cancers. Significant changes take place at the cellular level in NK cell activity with cancer cells as evidenced by the SERS profile. Intracellular changes in NK cells due to upregulation or downregulation of proteins are drivers of spectral changes. On observation of the SERS profile, the major contributing metabolites in breast cancer were disulfide stretching proteins (increased), tyrosine (decreased), phenylalanine (decreased), nucleic acids (decreased) corresponding to 521 cm^−1^, 854 cm^−1^, 1000 cm^−1^ and 1662 cm^−1^, respectively. These collective data show that NK cells associated with different cancer types are different (Fig. 7a–c).

The association of NK cells with tumor did not result in a homogenous signal as once thought of, rather they are uniquely heterogenous with distinct subsets. Despite the presence of minor differences, the principal component analysis (Fig. 6c) did not demonstrate clear clustering between cancer and non-cancer-cell-associated NK cells. The overlapping clusters can be possibly due to the immune escape phenomenon exhibited by cancer cells on association with NK cells resulting in the signals similar to the non-cancer-associated NK cell^70^. We observed a strong negative correlation on the dependence of NK cell SERS signature for diagnostic purpose with cancer cells (Fig. 6e). Since NK cells are preferentially cytotoxic to CSCs, we hypothesized that NK cells associated with CSCs could provide a better diagnostic signature when compared with non-cancer-associated NK cell.

### Evaluation of CSC-associated NK cell for cancer diagnosis

We compared the metabolic profile of CSC-associated NK-92 cells. The major changes in NK-CSCs metabolites were a significantly decreased disulfide stretching proteins, relative increase in quantity of nucleic acids, decrease in tyrosine and phenylalanine, increase in glycogen and fatty acids and lipids, increase in carotenoids, phospholipids, cytosine, decrease in tryptophan corresponding to 521 cm^−1^, 787 cm^−1^, 854 cm^−1^, 1000 cm^−1^, 1048 cm^−1^, 1137 cm^−1^, 1168 cm^−1^, 1268 cm^−1^, 1509 cm^−1^, 1339 cm^−1^. The decrease in the peak at 520 cm^−1^ of NK cell in association with CSCs indicate that NK-CSCs have regulated their Killer immunoglobulin receptor (KIR) expression, suggesting a possible decrease in MHC expression by CSCs. This paves way for increased cytotoxicity. The loss or decreased expression of MHC in CSCs is correlated with better clinical outcomes and a promising strategy to reverse the immune escape^71^. The most distinct bands typical of lymphocytes are seen at 1522 cm^−1^ and 1158 cm^−1^ belonging to carotenoids^59^. As mentioned earlier, carotenoids are robust Raman scatterers and have characteristic Raman spectra. Dramatic changes in the profile of CSC-associated NK cells indicated the suitability of CSC-associated NK cell for further analysis (Fig. 6e). One significant feature of this association is that unlike cancer cells, CSCs are unable to escape immune detection with NK cell and their association is readily reflected in their tumor profile. NK cells have a unique affinity for CSC as shown by many studies where NK cells lyse and kill CSC population in mice. The observed spectra show the immune cell stimulated/inhibited status upon interaction with the cancer cells. Several markers that are indicative of lymphocyte activation were identified from the spectra. The peak at 521 cm^−1^ is a disulfide band suggestive of the formation of immunoglobulins^72^. The disulfide bonds are characteristic in Raman spectra and appear distinct and separated from other peaks and help in the conformation of protein. NK cells exhibit immunoglobulin receptor protein on its surface KIR involved in the education of NK cell. A comparison of a heatmap of circulating NK cell and CSC-associated NK cell shows substantial differences in metabolic characteristics (Fig. 6f). Principal Component Analysis (Fig. 6g). show clear separation of clusters between CSC-associated NK cells and non-tumor-associated NK cells. K means clustering further confirmed the formation of clear clusters (Fig. 6h).

NK cells have a major function in tumor control in early stages and tumor progression in late stages. NK cells being the first line of defect against cancer, kill cancer cells without any prior sensitization. The NK cell also has a function in recruiting other immune cells into tumor microenvironment. Their ability to sense the presence of tumor is by recognizing the downregulation of MHC class molecule in stressed cells or tumor cells. NK cells preferentially kill these cells that show low expression of MHC class 1 receptor. NK cell mimic normal cell behavior when they interact with cancer cell (as seen with overlapping principal components) due to their downregulation of MHC class I receptor leading NK cells to believe that cancer cells are a part of self. This immune evasion is the hallmark of the cancer cell. On the other hand, NK cell on interaction with CSC show separate clustering. Therefore, we confirm that the use of CSC-associated NK cell would be ideal for diagnostic purposes. The differences in the CSC-associated NK cell and cancer-associated NK cell spectra are reproducible and significant (Fig. 7b, f and j) as seen with t-test. (*P* < 0.0001).

Principal component analysis demonstrated clear clustering of PC scores confirming substantial variation in the signature of breast, lung and colon CSC-associated NK cells compared to non-cancer-associated NK cells (Fig. 7c, g and K). Our results clearly demonstrate the high correlation of NK cells with CSCs and NK cell signatures reflecting the watchful vigilance of tumors by NK cells. Our results strongly show the plasticity of CSC-associated natural killer cells phenotypes with different cancer types of CSCs reflected in the circulation which can be useful in cancer diagnosis. Similar to NK-92 cells, SERS spectra of primary NK cells demonstrate a similar pattern on interaction with CSCs of breast, lung and colorectal (Supplementary Fig. 4c). Supplementary Figure 4d shows the principal component analysis demonstrating clear separation of clusters between CSCs and non-cancer cells using the spectra of primary NK cells cocultured with CSCs. This further established that even primary NK cells demonstrate CSC-specific activity.

We also looked at how tumor-associated NK cells in-vitro compared to the NK cells from real patients. As shown in Supplementary Figure 7a, the spectra of NK cells from cell culture and patient-derived NK cells demonstrated similar Raman shifts. (For breast cancer (Supplementary Fig. 7a), the wavenumbers for the peaks at 1001 cm^−1^, 1044 cm^−1^, 1121 cm^−1^, 1450 cm^−1^, 1606 cm^−1^, 2863 cm^−1^ and 2934 cm^−1^, for lung cancer (Supplementary Fig. 7c), the peaks at 975 cm^−1^, 1004 cm^−1^, 1044 cm^−1^, 1122 cm^−1^, 1313 cm^−1^, 1450 cm^−1^, 1521 cm^−1^, 1660 cm^−1^, 2858 cm^−1^, 2932 cm^−1^ were seen). As expected, there were variations in the intensities of some Raman shifts. Multivariate analysis (principal component analysis) was undertaken. The scatter plot of PC1 Vs PC2 Vs PC3 with principal component analysis demonstrated clustering of both types of cells in one single cluster for breast cancer as well as for lung cancer (Supplementary Fig. 7d, e). The cells will form separate clusters if there is discrimination observed in the cells with PCA. Therefore, we concluded that the evaluation of tumor-associated NK cells in culture and the NK cells of actual cancer patients demonstrated strong similarity to one another.

### Prediction of tumor-associated nk cells for cancer diagnosis directly with patient blood—without cellular isolation

**Fig. 8:**
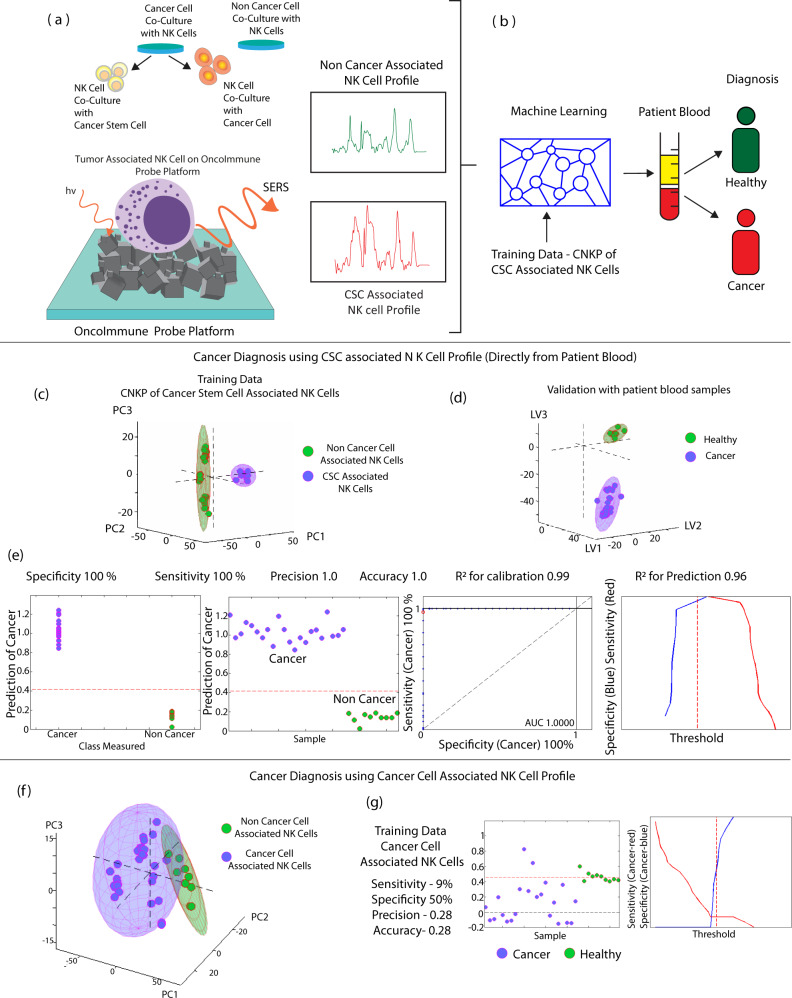
Prediction of tumor-associated NK cells directly with patient blood (without cellular isolation) using CNKP for cancer diagnosis. **a** Schematic representation of training data from cell culture (representative Raman spectra—non-cancer-associated NK cell -green, csc-associated NK cell—red). Source data are provided as Source Data File. **b** Schematic representation of liquid biopsy. **c** Prediction of tumor-associated NK cell directly from patient blood with CSC-associated NK cell profile using exploratory training data from csc-associated NK cells (**d**) validation with patient blood spectra. **e** Diagnosis of cancer with 100% sensitivity (red) & specificity (blue) (**f**) PCA of Raman spectra of non-cancer-cell-associated NK cells and cancer-cell-associated NK cells showing inability of cancer-cell-associated NK cell to provide accurate classification. **g** Sensitivity, specificity threshold analysis of training data demonstrates poor classification with cancer-cell-associated NK cell. Raman measurements were taken 10 times and averaged.

Next, we assessed the diagnostic accuracy of CNKP classification by collecting SERS spectra from 22 clinical samples obtained from Ontario Tumor Board (OTB). Peripheral blood from cancer patients diagnosed by clinical presentation and histopathological diagnosis was processed to extract the buffy coat layer by density gradient centrifugation. The patient cohort had three tumor types consisting of breast adenocarcinoma (*n* = 8), lung carcinoma (*n* = 7) and colorectal carcinoma (*n* = 7) as shown in Supplementary Table 4. 5 µL of buffy coat was dropped on the sensor and SERS spectra were taken at 785 nm wavelength. Partial least squares regression—discriminant analysis was done to discriminate healthy and tumor samples (Fig. 8b).

Briefly, PLSDA is a supervised machine learning analysis that identifies features that contribute to the most variation. The PLSDA models with cross validation was able to discriminate two clusters (*R*^2^ = 0.9) and showed excellent discriminatory power with 100% sensitivities and specificities and correctly predicted the samples as healthy or having cancer. We did a principal component analysis to find if the input data was discrete or not and subsequently the discrete dataset from NK cells associated with CSCs were used as training input data for the PLSDA analysis. For subsequent training of the algorithm, we used the patient’s buffy coat cohort as input for validation data. The results generated 100% accuracy, 100% precision, 100% specificity and 100% sensitivity and prediction of 1.0, Area under the curve = 1 with *R*^2^ for calibration as 0.99 and *R*^2^ for prediction as 0.96 (Fig. 8e). In contrast, the training data from NK cells associated with cancer cells during training with PLSDA yielded very poor prediction with accuracy of 28%, sensitivity of 9% and specificity of 50% showing the predictive power of NK cells associated with CSCs as compared to NK cells associated with cancer cells (Fig. 8g).

The molecular analysis of PLSDA provided basis for classification. As per Supplementary Fig. 8a, the Variable of importance scores (VIP scores) of vibrational peaks with the VIP score > = 1 were plotted. More number of peaks assigned to nucleic acids as well as proteins were evident in the VIP score plot. The loadings of latent variables also demonstrated that the peaks for nucleic acids, proteins as well as lipids were responsible for complete clustering without ambiguity for cancer vs non-cancer. This also demonstrated the critical features of holistic analysis of NK cell spectra for accurate cancer diagnosis.

### Localization of cancer using OncoImmune probe platform based CNKP directly with patient blood—without cellular isolation

**Fig. 9:**
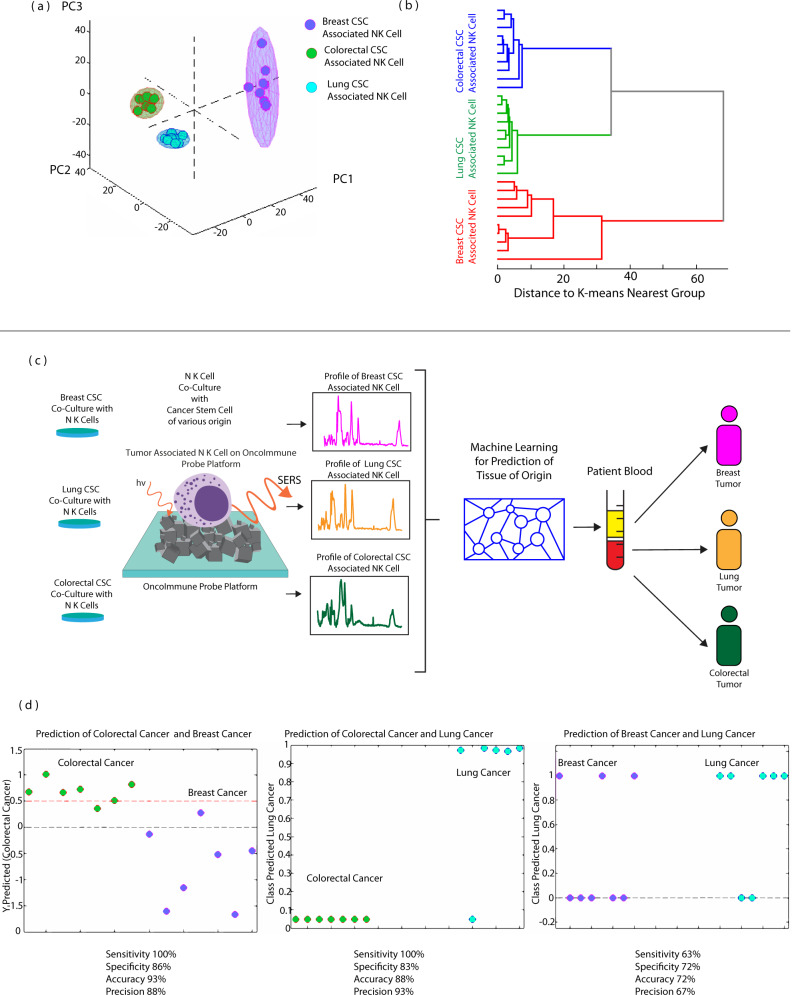
Localization of cancer with Raman profile of CSC-associated NK cell. Localization of cancer with Raman profile of CSC-associated NK cell. **a** Principal component analysis and (**b**) Hierarchical clustering analysis demonstrated clear clustering of breast, lung and colorectal CSC-associated NK cell. **c** Schematic representation of liquid biopsy. Representative Raman spectra of breast (pink), lung (orange), colorectal (green) CSC-associated NK cells. Source data are provided as Source Data File. **d** Classification between colorectal and breast cancer with 100% sensitivity & 86% specificity. Classification between colorectal and lung cancer with 100% sensitivity and 83% specificity. Classification between breast and lung cancer with 63% sensitivity and 72% specificity. Raman measurements were taken 10 times and averaged.

In addition to cancer diagnosis, tumor-associated NK SERS profile can distinguish healthy and patients with different types of cancer. Unsupervised principal component analysis and hierarchical cluster analysis can unambiguously discriminate healthy or non-cancer-associated NK cells and three individual tumor varieties i.e., breast, lung and colorectal (Fig. 9a, b) to give tumor specific SERS spectral wavenumbers that were used as training and validation data inputs for tissue of origin algorithms. Supplementary Figure 4e shows Primary NK cells having cancer-specific behavior. On co-culturing with CSCs of various tumor types, clear clustering was observed with principal component analysis. Supplementary Figure 9 shows signals of primary NK cells cocultured with CSC derived from cancer patients also confirmed the applicability of NK cells for cancer diagnosis. Identification of tumor location was possible with molecular analysis of tumor-associated NK (primary) as shown in Supplementary Fig. 10. Using MCR scores and SVM discriminative analysis of the tumor-associated NK cells, we identified sensitivity of 100%, specificity of 86%, accuracy 93% and precision of 88% for colorectal vs breast cancer and sensitivity of 100%, specificity of 83%, accuracy 88% and precision of 93% for colorectal vs lung cancer and sensitivity of 63%, specificity of 72%, accuracy 72% and precision of 67% for breast vs lung cancer allowing multilevel diagnosis across different tumors (Fig. 9d). An accuracy of 86% and 72% demonstrated satisfactory results in terms of diagnostic tool for cancer. The low specificity can be attributed to intra tumor variation. Such liquid biopsy will radically change the direction of cancer diagnosis for multiple types of cancer on validation in large cohort studies.

### Validation of the differential SERS expression seen in cancer and CSC interacted NK cells and healthy NK cells using RT-qPCR and glycolysis stress test

**Fig. 10:**
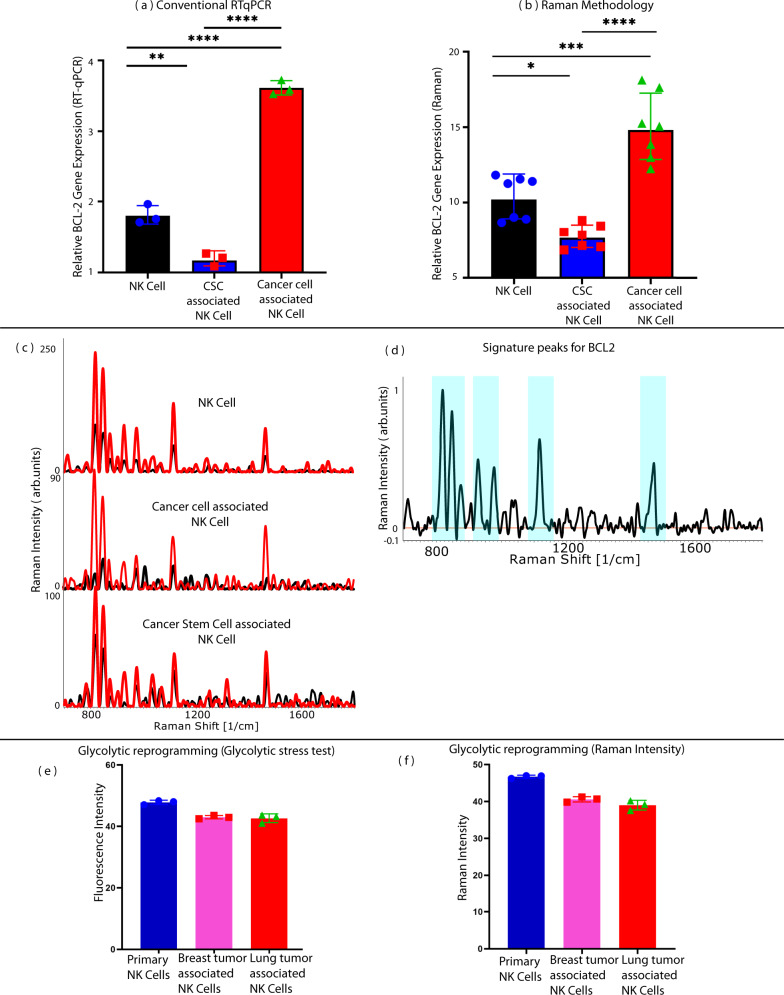
Validation of the differential SERS expression seen in cancer and CSC-associated NK cells and healthy NK cells using RT-qPCR and glycolysis stress test. **a** Conventional RT-qPCR results for quantification of BCL2, *n* = 3 biologically independent experiments, analysis of variance (ANOVA) between cancer-associated NK cells, CSC-associated NK cells and control NK cells. **b** Detection of BCL2 expression with Raman methodology demonstrating similar trend with ANOVA between cancer-associated NK cells, CSC-associated NK cells and control NK cells (*P ≤ 0.05, **P ≤ 0.01, ***P ≤ 0.001, ****P ≤ 0.0001). **c** Raman spectra of BCL2 amplified cDNA (red) and unamplified cDNA (black) (**d**) Difference spectra (black) generating signature Raman profile for BCL2. Source data are provided as Source Data File. **e** Glycolytic reprogramming quantification with glycolysis assay (**f**) Comparison of glycolytic reprogramming quantification with Raman intensity demonstrating similar trends. 20 biologically independent samples were used, and Raman measurements were taken 10 times and averaged. For gene expression analysis, three independent experiments were performed, and 7 independent experiments were performed for Raman.

To validate the proposed method of SERS to detect changes in NK cell signatures between cancer and CSC interacted NK cells and healthy NK cells, two studies were undertaken. First, we analyzed the relative mRNA expression levels of *BCL2* through RT-qPCR. *BCL2* (B cell lymphoma 2) gene encodes the *BCL2* family of regulatory proteins that is responsible for the regulation of apoptosis. *BCL2* influenced tumor-associated changes in immune cells favor the downregulation of pro-apoptotic proteins resulting in resistance to NK cell-mediated cell cytotoxicity^73^. The expression of *BCL2* is an activation/proliferation marker in the NK cells^74^. NK cells have increased expression of *BCL2*, favouring immune escape by the cancer cells. However, the trend is reversed in the presence of CSCs as NK cells have zero or reduced expression of *BCL2* favouring apoptosis and killing of cancer cells^74^. The results of RT-qPCR, following ANOVA (Analysis of variance), show that the expression of *BCL2* genes was significantly upregulated in NK cells cocultured with cancer cells compared to control NK cells (*P* < 0.0001). NK cells cocultured with CSCs showed significant downregulation of *BCL2* gene expression when compared to control NK cells (*P* < 0.001). Thus, there is a substantial difference between the expression levels of NK cells cocultured with cancer and CSCs. These results imply that CSC interacted NK cells are genotypically different from cancer interacted NK cells. Next, to compare the results of RT-qPCR with SERS, signature peaks of *BCL2* were identified, and Raman intensity was calculated, and compared with cancer, CSC interacted NK cells and healthy NK cells. As shown in Fig. 10a, b, the Raman experiment also demonstrated a similar trend of *BCL2* gene expression as the conventional RT-qPCR.

Second, NK cells have to compete with tumor cells in a nutrient-depleted environment for glucose and amino acids. NK cells, therefore, exhibit the ‘Immune Warburg phenomenon’ and metabolic switch to aerobic glycolysis^75–77^. In order to investigate the glycolytic reprogramming in CSC-associated NK cells, we undertook a glycolysis stress test for glycolytic characterization in live NK cells using glycolysis assay (ABCAM ab222946). Quantification of glycolysis was undertaken as per the manufacturer’s protocol. The signature peaks at 866 cm^−1^ and 835 cm^−1^, corresponding to lactate and pyruvate, respectively, were used to detect glycolytic reprogramming that gives rise to distinct Raman metabolic signature. The intensity of peaks for glycolysis reprogramming demonstrated the lowest intensity for lung-cancer-associated NK cells. The intensity was higher for breast-cancer-associated NK cells as compared to lung cancer, but it also showed much lower intensity as compared to control NK cells. The results of these observations are shown in Fig. 10e, f.

We have successfully demonstrated the feasibility of adopting signals reflecting changes in the metabolic profile of Natural Killer cell (NK cell) during tumor-associated NK cell activation, for cancer diagnosis. Profiling of tumor-associated circulating NK cell activity (CNKP) was introduced as a pathway for cancer diagnosis with liquid biopsy. Experimental demonstration of dissimilar profiles between cancer-associated NK cells and CSC-associated NK cells with statistical significance was achieved. This was instrumental in demonstration of the crucial function of CSC-associated NK cells in cancer diagnosis. Molecular signatures of CNKP were cancer-type specific. This property of tumor-associated NK cells was influential in the prediction of tumor location. SERS functionalized OncoImmune probe platform with small scaled probes was designed for this marker-free approach. We achieved ultralow concentrations (up to femtomolar 10^−15^M of analyte molecules). This was attributed to the configuration of probes by narrowing of the atomic scale probe apices, instrumental in the improvement of localized surface plasmon resonance. The 3D arrangements of the nanoprobes ensured NK cell trapping, critical in signal amplification and transmission. Highly reproducible signals were achieved. CNKP of three hard-to-detect cancer-cell lines (triple negative breast adenocarcinoma, small cell lung cancer, colorectal adenocarcinoma) was achieved with single-cell sensitivity. We were able to identify cancer from non-cancer using very small amount of peripheral blood (5 µL) with 100% prediction accuracy using machine learning model trained with SERS signals of NK cell activity in cell culture. Prediction of tumor location achieved prediction accuracy of up to 93%. This approach overcomes lack of patients by using data from easy to collect cell culture and by identifying the similarity of the features of NK cell activity in patient blood (without cell isolation) through machine learning. This method also provides a basis for classification. As cell culture data was used for training, multivariate analyses of cell-culture data provided an explainable basis. Such liquid biopsy using OncoImmune probe platform-based SERS has potential to radically change the direction of cancer diagnosis for multiple types of cancer on validation in large cohort studies. This work sets the stage in advancing the knowledge of tumor-associated NK cells and its use in liquid biopsy.

## Methods

### Clinical sample acquisition

This study was conducted in accordance with Ryerson Ethics Board of Ryerson University (REB 2020-275). Informed written consent and blood samples from cancer patients were obtained by Ontario Tumor Bank (OTB). The buffy coat was extracted by density gradient centrifugation. The Details on the clinical features, gender and demographic data are provided in Supplementary Table 4.

### Cell culture

The primary NK cells (Stem cell technologies) and NK-92 cell line (derived from non-Hodgkin lymphoma) were obtained from American Tissue Type Culture Collection (ATCC, USA) and maintained in Alpha minimum essential medium w/o nucleosides (catalog no 36453) with 0.2 mM myo-inositol, 0.1 mM 2-mercaptoethanol, 0.02 mM folic acid, 12.5% horse serum and 12.5% fetal bovine serum. To maintain sufficient proliferation IL-2 (catalog no 78036_c) was added at 150 IU/ml. Cancer-cell lines were similarly obtained from ATCC for breast adenocarcinoma (MDA-MB231), non-small lung carcinoma (H69 AR) and colon adenocarcinoma (Colo 205). Breast-cancer-cell line was maintained with DMEM with 10%FBS. The lung and colon cancer-cell lines were maintained in RPMI 1640 with 10% FBS. All cells were cultured at 37 C in 5% CO_2_ atmosphere.

### Synthesis of OncoImmune probe platform

Nickel sheets measuring 2 (w) × 12 (l) × 0.019 (h) inches were purchased from McMaster-Carr. The sheets were cut into 2 (l) × 1.5(w) inches to fit in the laser system. The nickel sheets were supported on stand for laser ablation. Femtosecond, Yb-doped fiber amplified; Clark MXR IMPULSE laser was used for the synthesis of nanoparticles. The following parameters were used: laser pulse width—214 fs, repetition rate—25 MHz, laser wavelength—1030 nm and power—14 W. EzCAD2 software was used to design the ablation area. The design of the ablation from the software transferred to the nickel sheet through piezoelectric scanner, with a scanning speed of 1 mm/sec. Nitrogen gas and oxygen gas were introduced separately in the ablation area using six Masterflex tubes secured on square bracket. The pressure was kept at 2 bar. The laser pulse interacts with nickel substrate at 90 degrees and the ablation plume interacted with the background nitrogen gas. The diagnostic probes then deposit on the substrate.

### Co-culture assays

NK-92 cells were cocultured with three cancer-cell lines using transwell apparatus (0.4 μm pore size, corning, Lowell, MA). NK-92 cells were seeded in the upper insert of 24 well transwell plate. The cancer-cell lines were seeded in three separate lower chambers. CSCs were grown separately prior to the assay in a serum-free media using ultralow low attachment plates. Tumor spheroids were verified in microscope and seeded in a set of other low chambers. After 24 h of co-culture, the NK-92 cells were centrifuged and resuspended in water for SERS analysis. NK cells grown separately without co-culture with cancer-cell lines were used as control.

### RT-qPCR

RNA was extracted from the cell pellets by the Trizol /chloroform (Invitrogen) extraction method and resuspended in diethylpyrocarbonate (DEPC)-treated water. RNA concentrations and ratios were determined photometrically (Nanodrop). The expression levels of BCL2 were assessed by RT-qPCR using one step PCR (New England Biolab E3005G NEB). Primer sequence Forward GGATGCTTTAT and reverse GCTTTATTTCATGAG. RT-qPCR reactions were performed on 96-well plates (Micro Amp® Fast Optical 96-well reaction plate with barcode; ABI, Foster City, CA, USA). The relative expression levels of target genes were expressed as fold-change against housekeeping gene GAPDH. Statistical analysis (one-way ANOVA) was done using Prism graphpad (v.9.2.0) compare the gene expression levels.

### Raman data acquisition

5 μl of buffy coat (blood) and 10 μl of cultured NK-92 cells were dropped on the OncoImmune probe platform. After 1 min (to allow trapping of NK-92 cells in the sensor mesh), Raman spectral scanning was done at 785 nm wavelength. A minimum of 10 spectra and 3 acquisitions were made with laser power at 5 W. The spectra were collectively saved and processed using Spectragryph software (V.1.2.9).

### Data analysis

The NK-92 cell-culture data were given as training data and the spectra from buffy coat were given as validation data. Data was collected in Excel files and Partial least squares regression analysis was done after doing multivariate analysis. PLSDA analysis was done using PLS Toolbox software (SOLO V 9.0). For the localization of cancer, data from buffy coat of known patient samples were used to train the algorithm. The statistical analysis for all the results is indicated in the figure legends. All data are represented as mean ± S.D. For SERS analysis two tailed Student’s *t* test was performed. *P* < 0.05 was considered significant. Experimental results were done at least three times, unless stated otherwise in figure legends.

### Reporting summary

Further information on research design is available in the Nature Research Reporting Summary linked to this article.

## Supplementary information

Supplementary Information

Reporting Summary

## Data Availability

Data supporting the findings of this study are available from the corresponding author upon reasonable request.  Source data are provided with this paper.

## Source data

Source Data
