# Supplementary material for: Minimally invasive detection of cancer using metabolic changes in tumor-associated natural killer cells with Oncoimmune probes
Source: Nat Commun. 2022 Aug 4;13:4527. doi: 10.1038/s41467-022-32308-x (PMC9352900; doi:10.1038/s41467-022-32308-x)
Supplement: Supplementary file 1 — Supplementary Information [file 41467_2022_32308_MOESM1_ESM.pdf]

Minimally Invasive Detection of Cancer Using Metabolic Changes in Tumor-Associated Natural Killer Cells with OncoImmune Probes

SUPPLEMENTARY INFORMATION

Deeptha Ishwar [1,2,3,4,6] , Rupa Haldavnekar [1,2,3,4,6], Krishnan Venkatakrishnan [1,2,4,5,7,] Bo Tan [1,3,5]

[1] Institute for Biomedical Engineering, Science and Technology (I BEST), Partnership between Ryerson University and St. Michael's Hospital, Toronto, Ontario M5B 1W8, Canada

[2] Ultrashort Laser Nanomanufacturing Research Facility, Faculty of Engineering and Architectural Sciences, Ryerson University, 350 Victoria Street, Toronto, ON M5B 2K3, Canada

[3] Nano Characterization Laboratory, Faculty of Engineering and Architectural Sciences, Ryerson University, 350 Victoria Street, Toronto, Ontario M5B 2K3, Canada.

[4] Nano-Bio Interface facility, Faculty of Engineering and Architectural Sciences, Ryerson University, 350 Victoria Street, Toronto, ON M5B 2K3, Canada.

[5] Keenan Research Center for Biomedical Science, Unity Health Toronto, Toronto, Ontario, M5B 1W8, Canada

[6] Deeptha Ishwar and Rupa Haldavnekar contributed equally.

[7] Corresponding Author: E-Mail: venkat@ryerson.ca

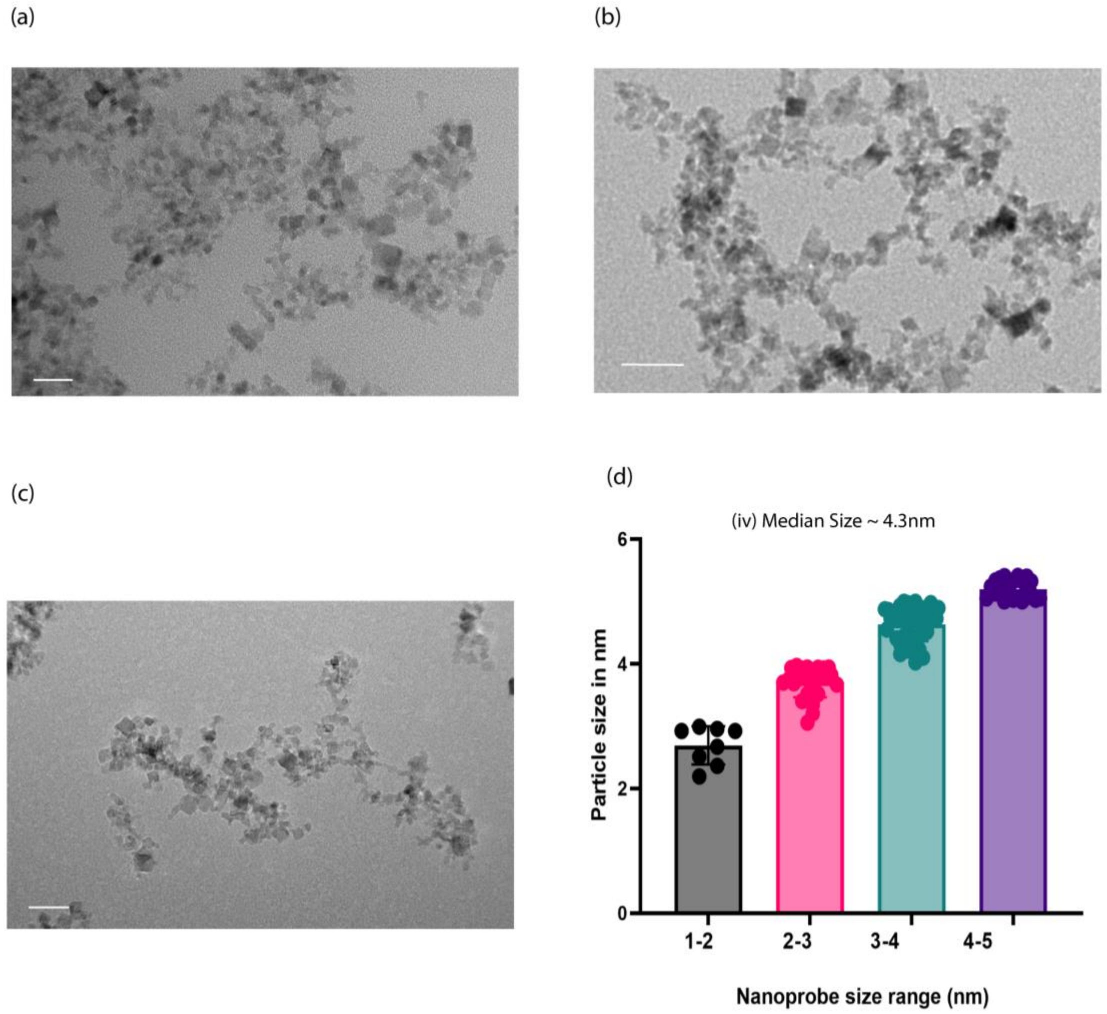

Supplementary Figure 1 : Charecterization of OncoImmune probe platform with Transmission Electron Microscopy. a, b, c - TEM images of OncoImmune Sensor showing nano scaled probes with sharp apices favouring excellent SERS signalling. Scale bar = 20 nm. d - bar chart of particle size distribution. Error bars show Standard Deviation.

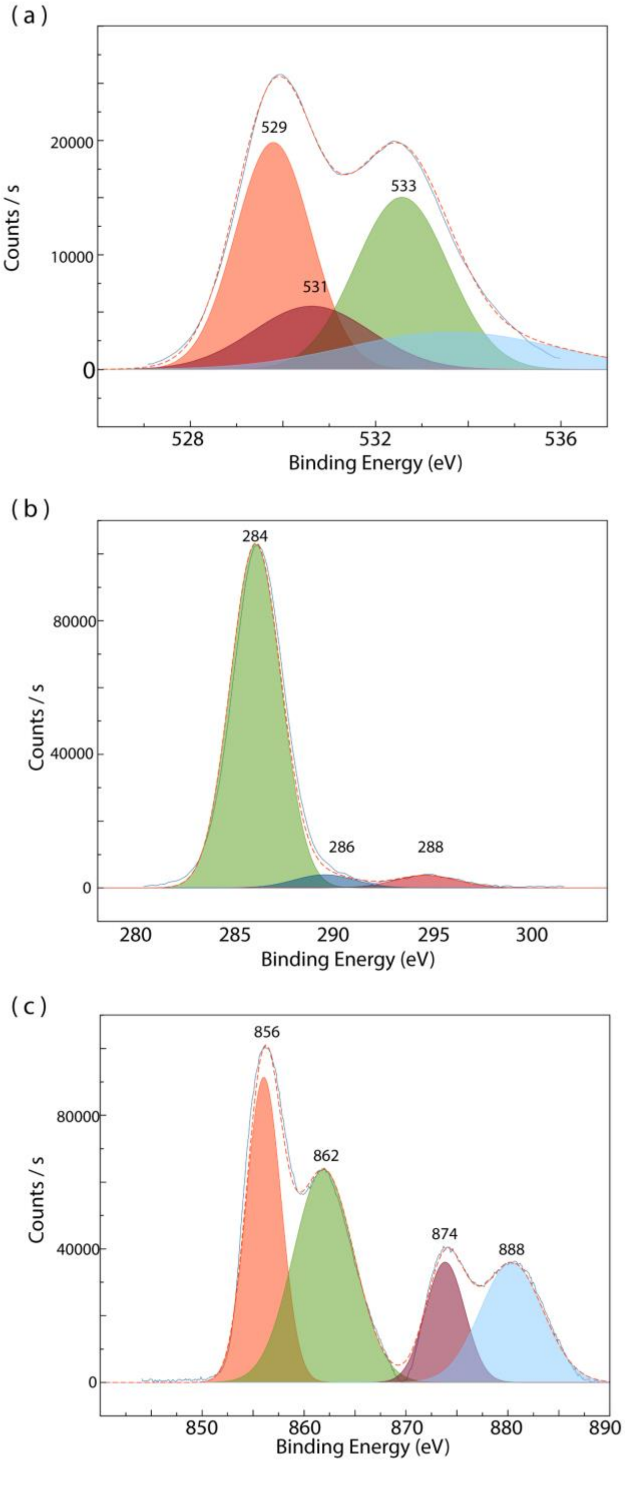

Supplementary Figure 2- Charecterization of OncoImmune probe platform with XPS analysis – (a) deconvoluted spectra of O1S showing presence of oxygen vacancies (b) C1S deconvoluted spectra and (c) Ni 2P deconvoluted spectra showing presence of nickel oxide. Source data are provided as Source Data File.

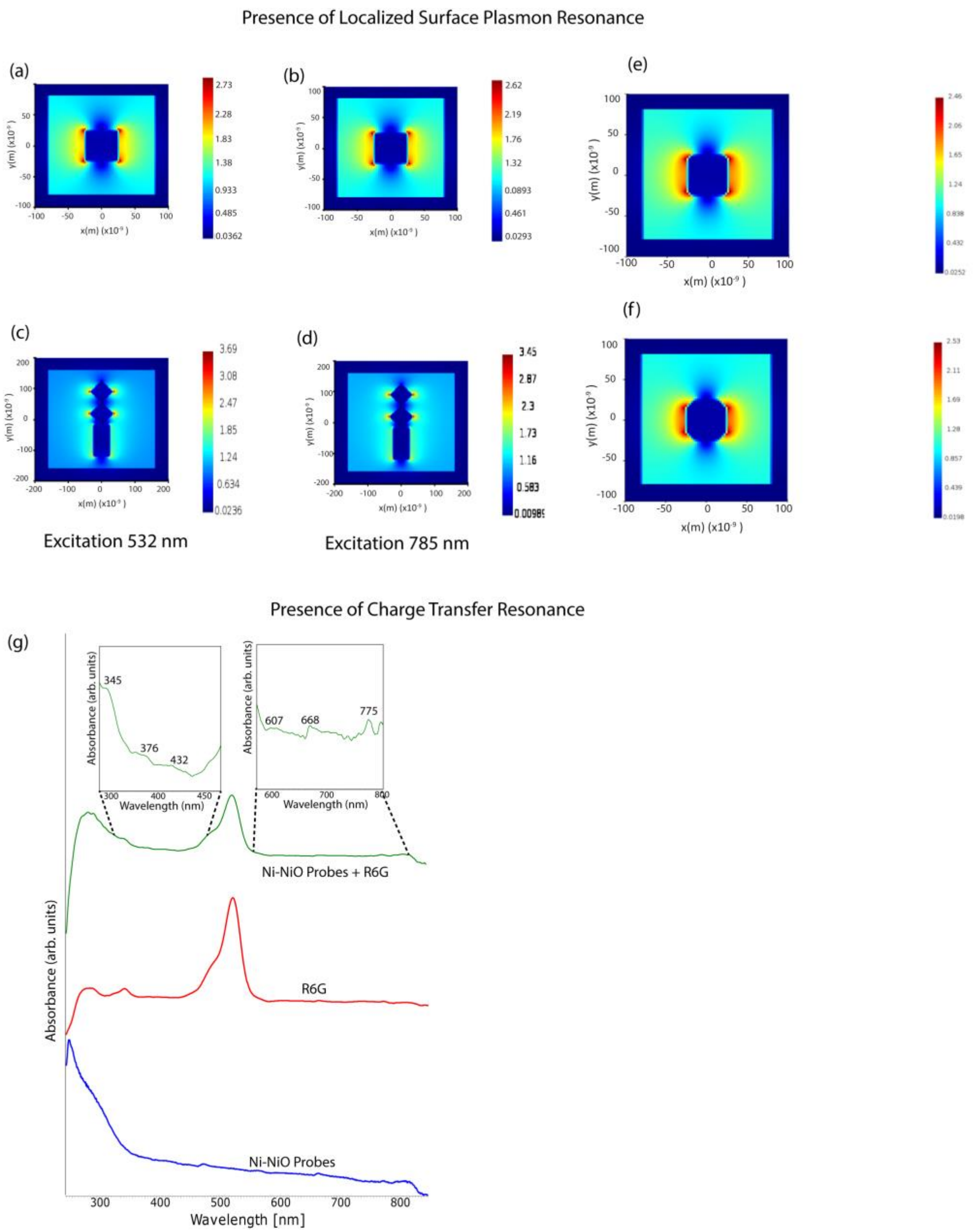

Supplementary Figure 3: OncoImmune probe platform demonstrating presence of surface plasmon and charge transfer resonance. Top panel showing FDTD simulation of Ni probes demonstrating the presence of localized surface plasmon resonance. (a) Electrical field enhancement at the apex of cubical probe calculated to be 2.73 eV at 532 and (b) Electrical field enhancement at the apex of cubical probe calculated to be 2.62 eV at 785 nm. (c) and (d) Substantial increase in the intensity of LSPR with presence of hot spots with multiple probes at 532 and 785 nm respectively. (e) and (f) FDTD simulation of probes with blunt apices and probes with round apices respectively. Reduction in the electric field at the probe apices as a result of more rounding of the probe apices can be seen. (g) UV-VIZ analysis with only Ni-NiO probes (blue), only R6G (red) and Ni-NiO

probes with R6G (green). The peaks in the green spectra at 345nm, 376nm, 432 nm, 607 nm, 668nm and 775 nm confirmed the presence of charge transfer mechanism. Source data are provided as Source Data File.

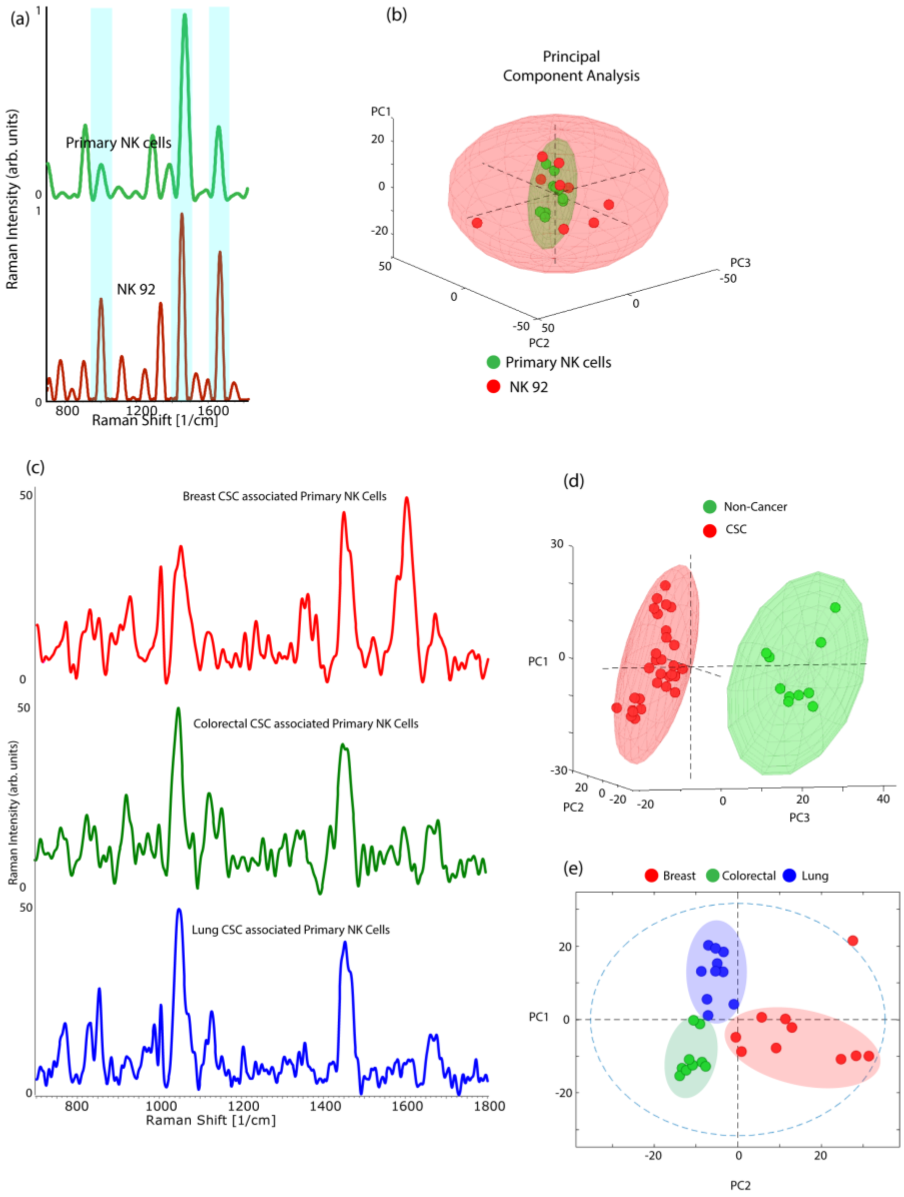

Supplementary Figure 4 - NK 92 and Primary NK cells demonstrate similar features – (a) Spectral similarity between NK92 (red) and Primary NK cells (green) (b) PCA demonstrated single clustering confirming similarity between NK 92 and Primary NK cells (c) Lung CSC (blue) , colorectal CSC (green) and breast CSC (red) associated Primary NK cell spectra (d) Applicability of Primary NK cells for Cancer Diagnosis with cancer stem cell (CSC) and non-cancer associated NK cell (e) Applicability of Primary NK cells for localization of cancer.

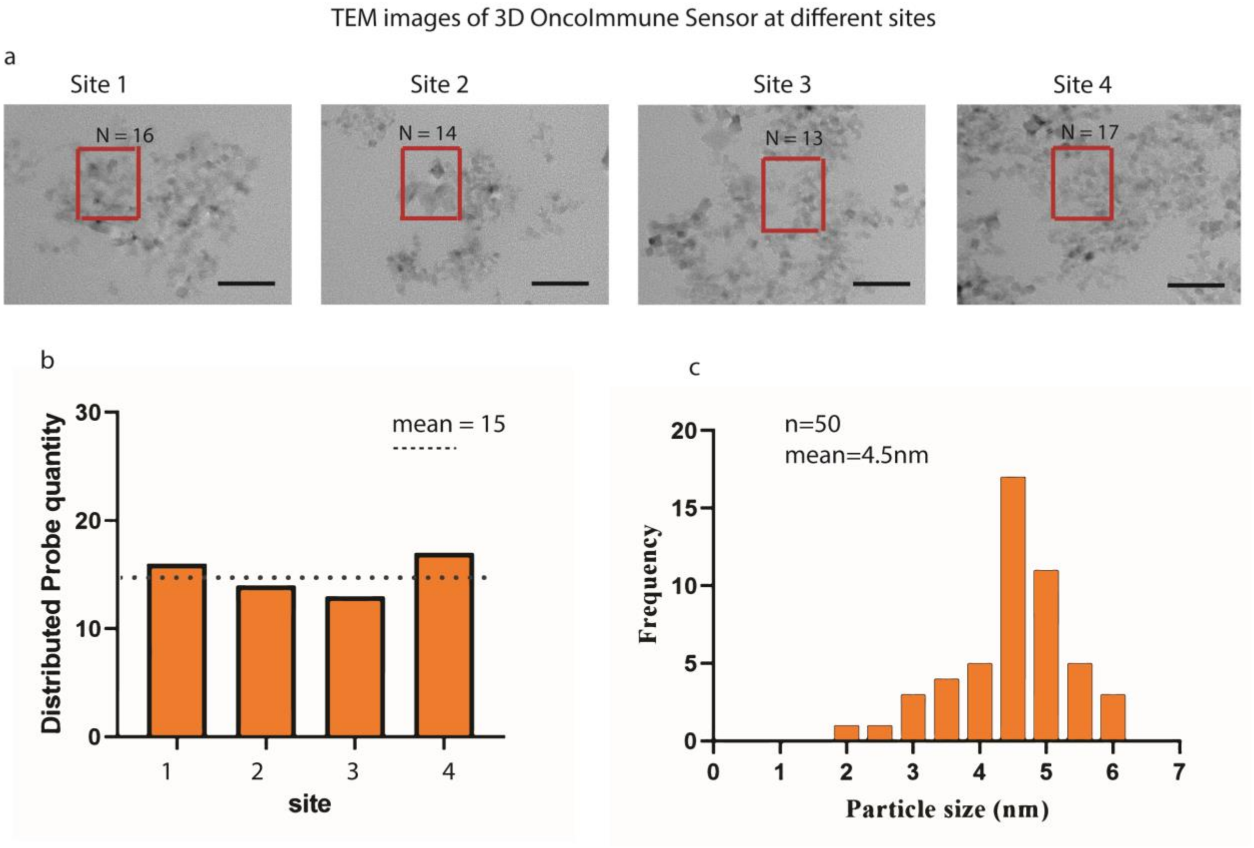

Supplementary Figure 5: Reproducibility of nanoparticle synthesis- (a) Quantification of probe at different sites using TEM images of 3D Oncoimmune sensor showing uniform production of probe during femtosecond laser synthesis. Scale bar = 20 nm. (b) distributed probe quantity at site 1 (16), 2 (14), 3 (13) and site 4 (17) with mean distribution of 15 particles. (c) - histogram showing particle size distribution with mean particle size of 4.5 nm. Source data are provided as Source Data File.

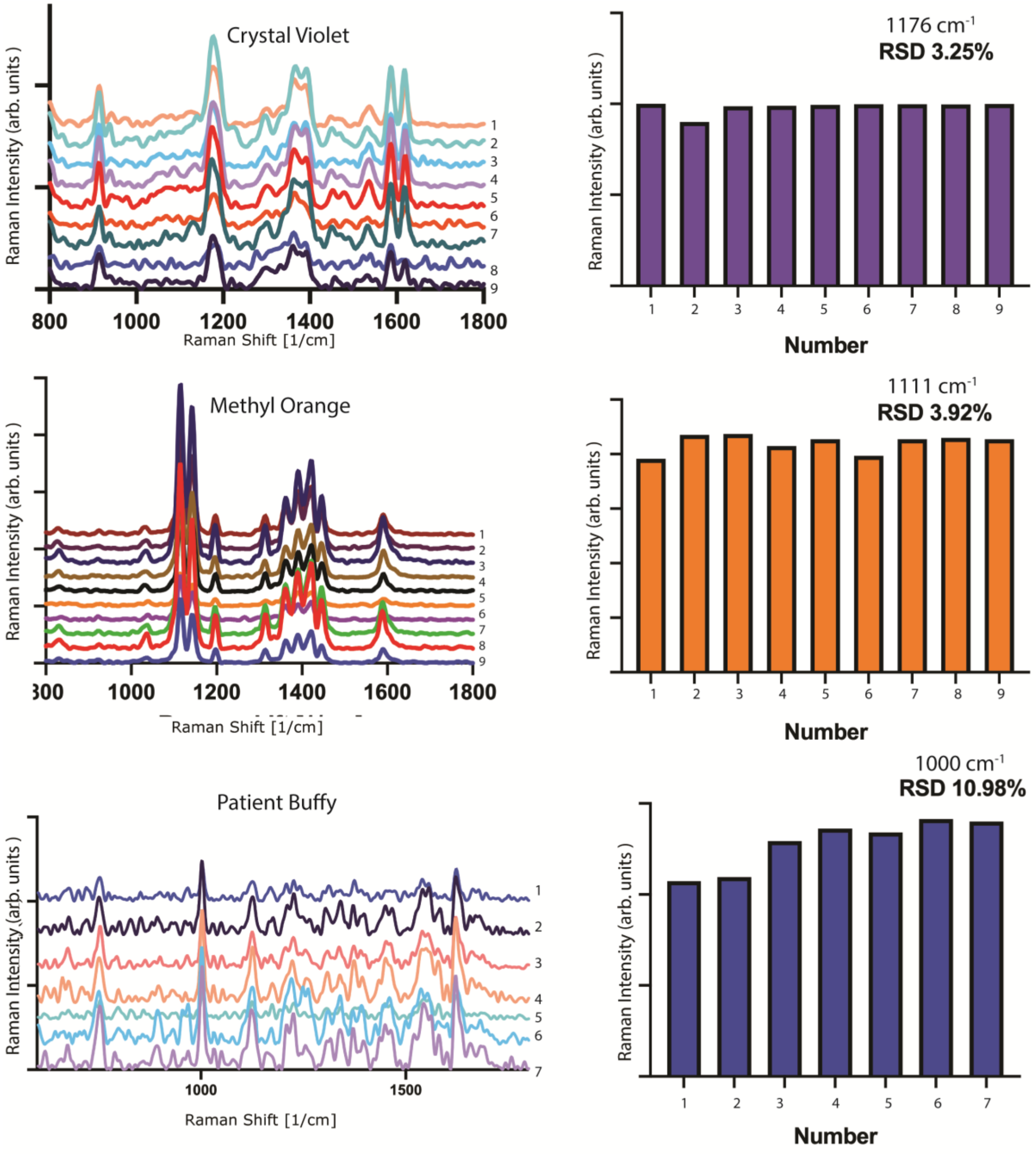

Supplementary Figure 6: Reproducibility of Raman spectra with OncoImmune probe platform– Left panel - Raman spectra of crystal violet, methyl orange and patient buffy coat. Right panel shows relative standard deviation for signature peak - crystal violet (1176 cm^{-1}), Methyl orange (111 cm^{-1}) and from patient's buffy coat (1000 cm^{-1}). Source data are provided as Source Data File.

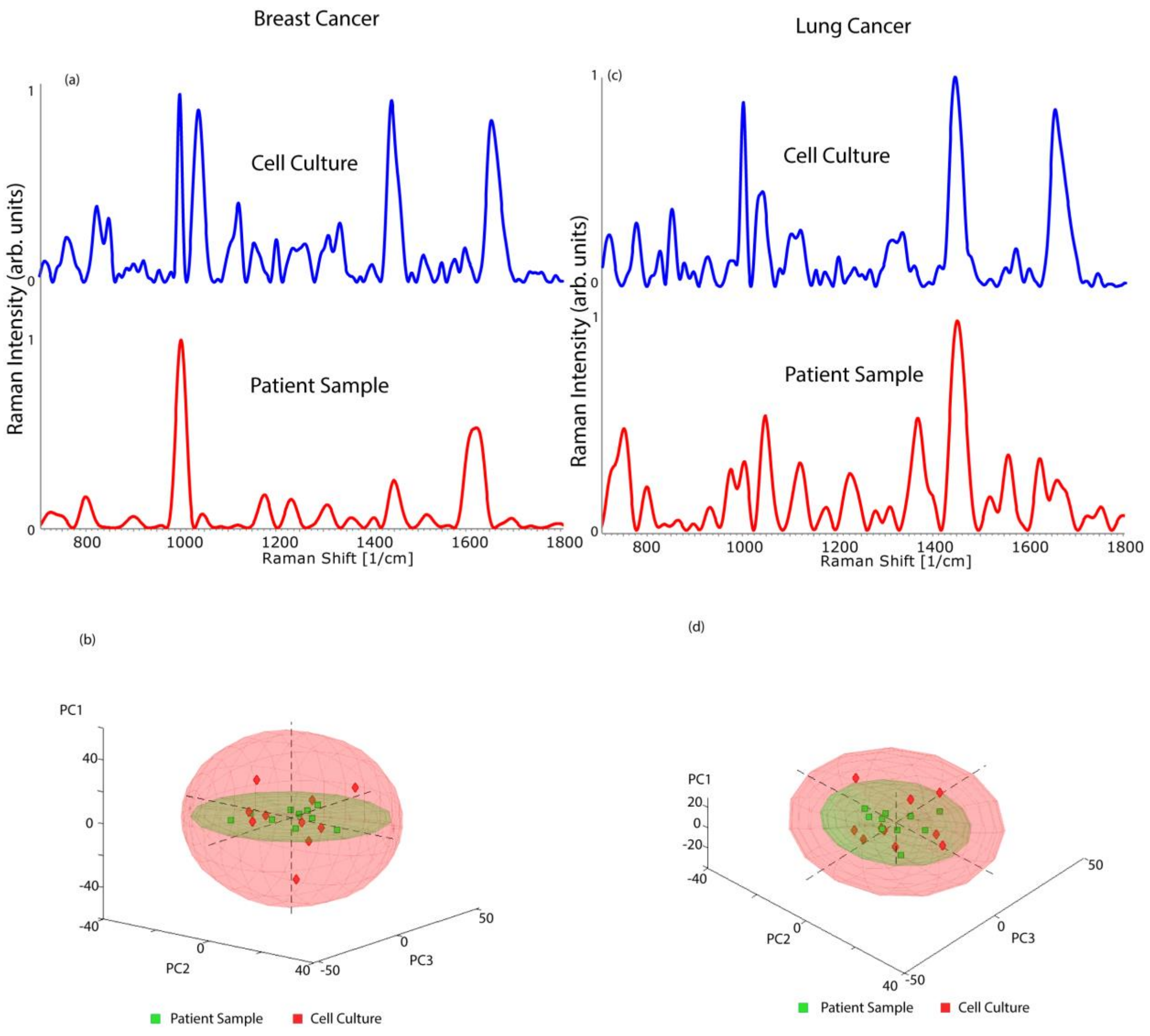

Supplementary Figure 7 – Similarity between patient NK cells (red) and cell culture NK cells (blue) (a) Raman spectra of NK cell associated with breast cancer in cell culture and patient samples. (b) Principal component analysis demonstrating similarity in cell culture and patient samples. (c) Raman spectra of NK cell associated with lung cancer in cell culture and patient samples. (d) Principal component analysis demonstrating similarity in cell culture and patient samples. Source data are provided as Source Data File.

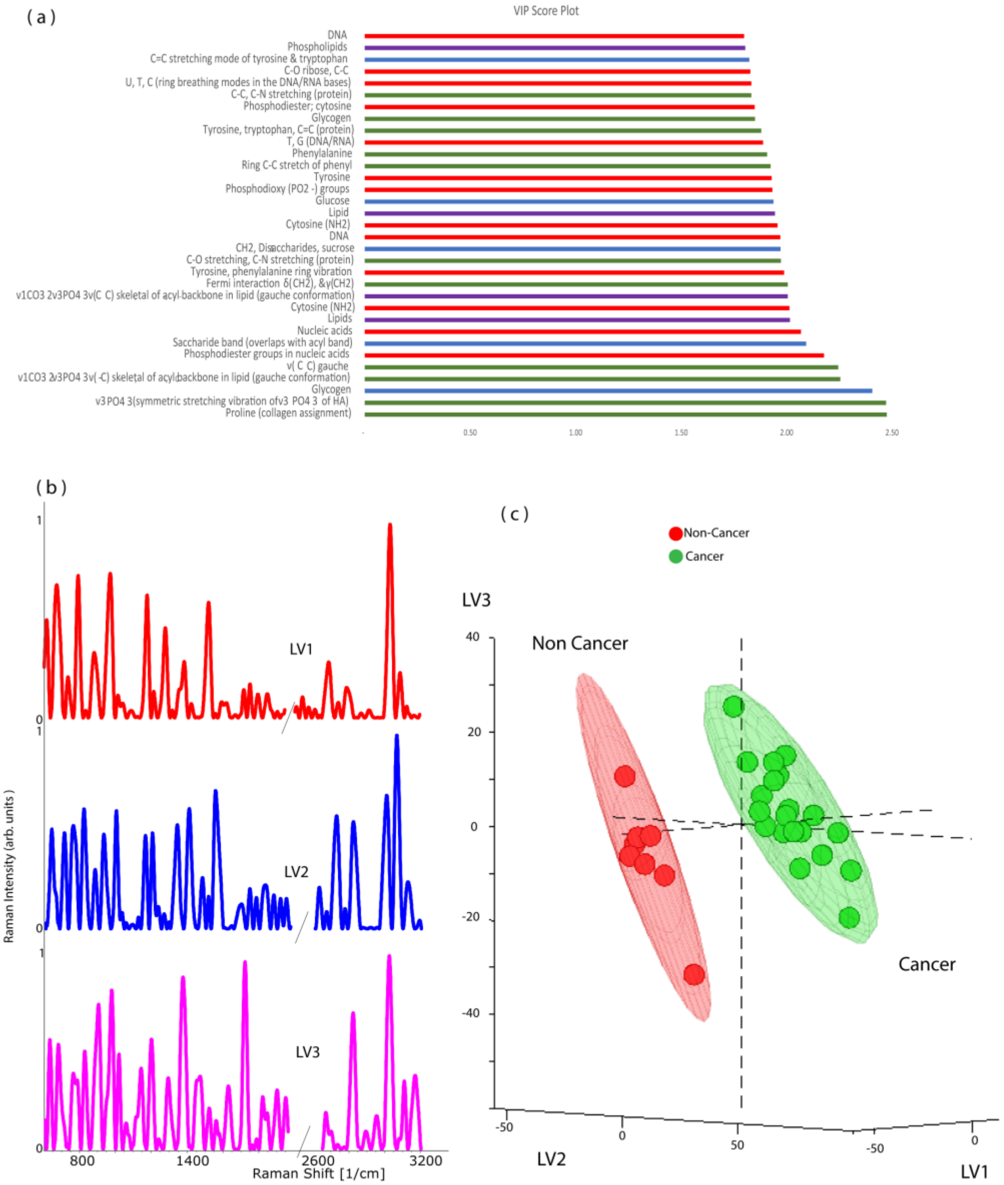

Supplementary Figure 8- Molecular analysis of Prediction of Tumor Associated NK Cells for Cancer Diagnosis – (a) VIP score plot with Raman assignments (b) Loadings of latent variables of PLSDA analysis LV1(red) LV2 (blue) and LV3 (pink) (c) Scatter Plot of LV1 vs LV2 Vs LV3 demonstrating clear clustering between cancer and non-cancer. Source data are provided as Source Data File.

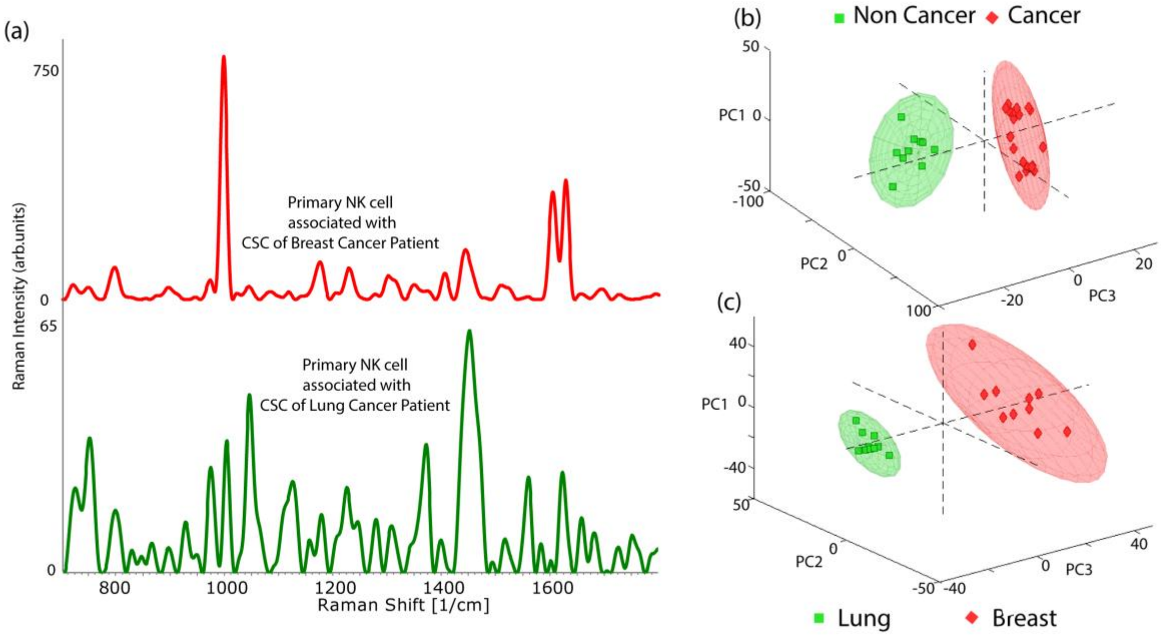

Supplementary Figure 9 – Applicability of primary NK cells with CSC from Patient samples- (a) Raman spectra of Primary NK cells co-cultured with CSC derived from Breast cancer (red) and Lung cancer patients (green) (b) Principal component analysis demonstrated clustering between cancer and non-cancer (c) Identification of cancer location with NK cell spectra demonstrated cancer specific behaviour. Source data are provided as Source Data File.

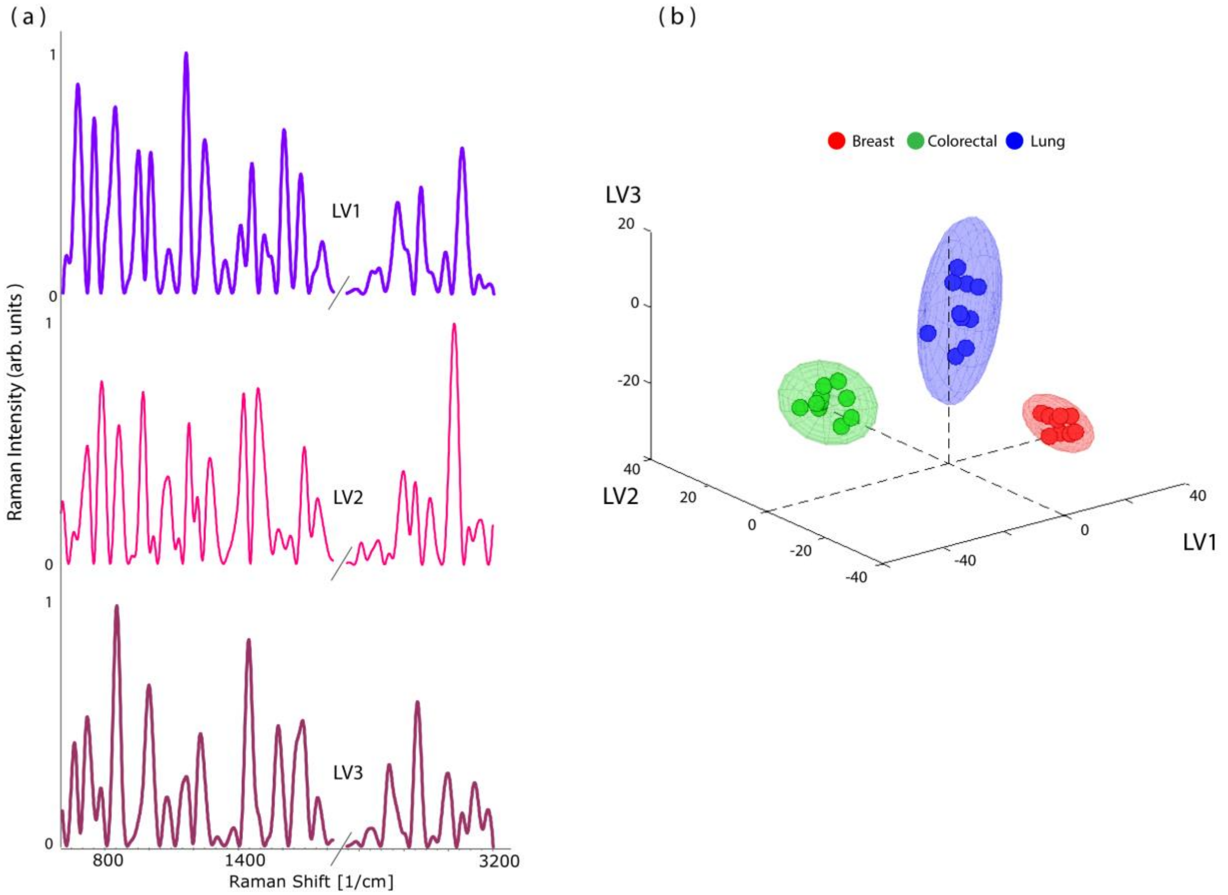

Supplementary Figure 10 - Molecular analysis of Prediction of Tumor Associated NK Cells for Tumor Location – (a) Loadings of latent variables of PLSDA analysis LV1 (purple) LV2 (pink) and LV3 (maroon) (b) Scatter Plot of LV1 vs LV2 Vs LV3 demonstrating clear clustering between different locations of tumor

Supplementary Table 1- XPS Spectra of OncoImmune Sensor

C1S Spectra

Peak Position	HWHM	Amplitude	Area
288	0.87	3781	6977
286	0.83	3946	7045
284	0.69	102888	152989

O1S Spectra

Peak Position	HWHM	Amplitude	Area
529	0.95	19829	40516
531	1.49	5520	17553
533	1.18	15027	37916
534	2.62	3263	18207

Ni2p Spectra

Peak Position	HWHM	Amplitude	Area
856	1.99	387105	91364
862	3.49	63706	474674
874	2.25	36048	173426
880	3.6	35884	275609

Supplementary Table 2- Raman Assignments for Rhodamine 6G[1,2]

Raman shift (cm^{-1})	Assignments
612	in plane bending of C-C-C ring
773	out of plane bending of C-H
1126	in plane bending C-H
1313	aromatic stretching of C-C
1363	aromatic stretching of C-C
1513	aromatic stretching of C-C
1651	aromatic stretching of C-C

Supplementary Table 3- Raman Assignments reported in this study[3–13]

Raman Shift (cm^{-1})	Assignments
521	disulphide bonds (S-S)
787	nucleic acids
830 & 854	tyrosine doublets
1001-4	phenylalanine
975	Ribose vibration, one of the distinct RNA modes
1044	n PO32(symmetric stretching vibration of n PO32 of HA
1048	glycogen
1121-2	nsym(C-O-C) (polysaccharides)
1158	carotenoids
1137	fatty acids
1044	v3PO3-4 (symmetric stretching vibration of v3PO3-4
1168	lipids
1268	carotenoids
1313	CH3CH2 twisting mode of collagen /lipid
1337	amide III
1339	tryptophan
1340	tryptophan
1450	CH deformation
1509	phospholipids
1521-2	carotenoids
1555	amide II
1606	Tyrosine, phenylalanine ring vibration
1660	Amide I (protein)
1662	nucleic acids
2858	CH 2 asymmetric stretch of lipids and proteins
2863	CH 2 symmetric stretch of lipids & CH 2 asymmetric stretch of lipids and proteins
2932	CH 2 asymmetric stretch
2934	CH 2 asymmetric stretch

Supplementary Table 4: Clinical and pathological details of breast, lung, and colorectal cancer patients.

Sample	Age (years)	sex	Site of primary	Histology	Grade/ Differentiation	Pathological T	Pathological N	Metastasis
B1	85-89	F	BREAST	Invasive ductal carcinoma	III	T2	N1a	M0
B2	50-54	F	BREAST	Invasive ductal carcinoma	III	T3	N3a	M0
B3	65-69	M	BREAST	Invasive ductal carcinoma	III	T3	N1	M0
B4	45-49	F	BREAST	Invasive ductal carcinoma	III	T2	N1a	M0
B5	85-89	F	BREAST	Infiltrating lobular	III	T3	N1a	M0
B6	55-59	F	BREAST	Invasive ductal carcinoma	III	T2	N1a	M0
B7	65-69	F	BREAST	Invasive ductal carcinoma	III	T2	N1a	M0
B8	50-54	F	BREAST	Invasive ductal carcinoma	III	T3	N2a	M0
L1	65-69	F	LUNG	Squamous carcinoma	III	T1c	N0	M0
L2	75-79	M	LUNG	Squamous carcinoma	III	T3	N0	M0
L3	60-64	M	LUNG	Squamous carcinoma	III	T2a	N0	M0
L4	70-74	F	LUNG	Adenocarcinoma	III	Tx	Nx	Mx
L5	55-59	F	LUNG	Acinar Adenocarcinoma	II	T2a	N0	M0
L6	60-64	F	LUNG	Acinar Adenocarcinoma	II	T2a	N0	M0
L7	55-59	F	LUNG	Acinar Adenocarcinoma	II	T2a	N0	M0
C1	50-54	M	COLON	Adenocarcinoma	IV	T3	N2b	M0
C2	45-49	F	COLON	Adenocarcinoma	II	T4	N1b	M1
C3	50-54	M	RECTUM	Adenocarcinoma	II	T3	N0	M0
C4	80-84	M	COLON	Mucinous (colloid) Adenocarcinoma	II	T3	N1a	M0
C5	85-89	F	COLON	Mucinous (colloid) Adenocarcinoma	II	T4	N2a	M0
C6	75-79	M	COLON	Adenocarcinoma	II	T2	N0	M0
C7	40-44	M	COLON	Adenocarcinoma	II	T4	N2b	M1

B-Breast, L-Lung, C-Colon, F-Female, M-Male, II-Moderate differentiation, III- poorly differentiated, IV-undifferentiated, T1c – tumor is greater than 2cm and less than or equal to 3cm. T2- Tumor is more than 3 cm but not more than 5 cm across. T2a- tumor size is between 3cm and 4cm. T3-Tumor size is more than 5cm and not more than 7 cm. T4a- Tumor is greater than 7cm. N0- No nodal metastasis, Nx- regional lymph node cannot be accessed. N1- metastasis in 1-3 regional lymph nodes. N2- metastasis in four or more regional lymph nodes. a-ipsilateral nodes, b- bilateral nodes, Mx- Distant metastasis cannot be accessed. M0- No metastasis, M1- Distant metastasis.

Supplementary References

1. Jensen, L. & Schatz, G. C. Resonance Raman scattering of rhodamine 6G as calculated using time-dependent density functional theory. *The Journal of Physical Chemistry A* **110**, 5973–5977 (2006).
2. He, X. N. *et al.* Surface-enhanced Raman spectroscopy using gold-coated horizontally aligned carbon nanotubes. *Nanotechnology* **23**, 205702 (2012).
3. Movasaghi, Z., Rehman, S. & Rehman, I. U. Raman spectroscopy of biological tissues. *Applied Spectroscopy Reviews* **42**, 493–541 (2007).
4. De Gelder, J., De Gussem, K., Vandenabeele, P. & Moens, L. Reference database of Raman spectra of biological molecules. *Journal of Raman Spectroscopy: An International Journal for Original Work in all Aspects of Raman Spectroscopy, Including Higher Order Processes, and also Brillouin and Rayleigh Scattering* **38**, 1133–1147 (2007).
5. Wesełucha-Birczyńska, A., Kozicki, M., Czepiel, J. & Birczyńska, M. Raman micro-spectroscopy tracing human lymphocyte activation. *Analyst* **138**, 7157–7163 (2013).
6. Stone, N., Kendall, C., Smith, J., Crow, P. & Barr, H. Raman spectroscopy for identification of epithelial cancers. *Faraday discussions* **126**, 141–157 (2004).
7. Naumann, D. Infrared and NIR Raman spectroscopy in medical microbiology. in vol. 3257 245–257 (SPIE, 1998).
8. Shetty, G., Kendall, C., Shepherd, N., Stone, N. & Barr, H. Raman spectroscopy: elucidation of biochemical changes in carcinogenesis of oesophagus. *British journal of cancer* **94**, 1460–1464 (2006).
9. Cheng, W., Liu, M., Liu, H. & Lin, S. Micro-Raman spectroscopy used to identify and grade human skin pilomatrixoma. *Microscopy research and technique* **68**, 75–79 (2005).
10. Malini, R. *et al.* Discrimination of normal, inflammatory, premalignant, and malignant oral tissue: a Raman spectroscopy study. *Biopolymers: Original Research on Biomolecules* **81**, 179–193 (2006).
11. Chan, J. W. *et al.* Micro-Raman spectroscopy detects individual neoplastic and normal hematopoietic cells. *Biophysical journal* **90**, 648–656 (2006).
12. Binoy, J. *et al.* NIR-FT Raman and FT-IR spectral studies and ab initio calculations of the anti-cancer drug combretastatin-A4. *Journal of Raman Spectroscopy* **35**, 939–946 (2004).
13. Puppels, G., Garritsen, H., Kummer, J. & Greve, J. Carotenoids located in human lymphocyte subpopulations and natural killer cells by Raman microspectroscopy. *Cytometry: The Journal of the International Society for Analytical Cytology* **14**, 251–256 (1993).
