# Supplementary material for: Minimally invasive detection of cancer using metabolic changes in tumor-associated natural killer cells with Oncoimmune probes
Source: Nat Commun. 2022 Aug 4;13:4527. doi: 10.1038/s41467-022-32308-x (PMC9352900; doi:10.1038/s41467-022-32308-x)
Supplement: Supplementary file 2 — Reporting Summary [file 41467_2022_32308_MOESM2_ESM.pdf]

nature portfolio

Corresponding author(s): Krishnan Venkatakrishnan

Last updated by author(s): May 31, 2022

Reporting Summary

Nature Portfolio wishes to improve the reproducibility of the work that we publish. This form provides structure for consistency and transparency in reporting. For further information on Nature Portfolio policies, see our Editorial Policies and the Editorial Policy Checklist.

Statistics

For all statistical analyses, confirm that the following items are present in the figure legend, table legend, main text, or Methods section.

n/a	Confirmed	
☐	☒	The exact sample size (*n*) for each experimental group/condition, given as a discrete number and unit of measurement
☐	☒	A statement on whether measurements were taken from distinct samples or whether the same sample was measured repeatedly
☐	☒	The statistical test(s) used AND whether they are one- or two-sided<br>*Only common tests should be described solely by name; describe more complex techniques in the Methods section.*
☐	☒	A description of all covariates tested
☒	☐	A description of any assumptions or corrections, such as tests of normality and adjustment for multiple comparisons
☐	☒	A full description of the statistical parameters including central tendency (e.g. means) or other basic estimates (e.g. regression coefficient) AND variation (e.g. standard deviation) or associated estimates of uncertainty (e.g. confidence intervals)
☐	☒	For null hypothesis testing, the test statistic (e.g. *F*, *t*, *r*) with confidence intervals, effect sizes, degrees of freedom and *P* value noted<br>*Give P values as exact values whenever suitable.*
☒	☐	For Bayesian analysis, information on the choice of priors and Markov chain Monte Carlo settings
☐	☒	For hierarchical and complex designs, identification of the appropriate level for tests and full reporting of outcomes
☒	☐	Estimates of effect sizes (e.g. Cohen's *d*, Pearson's *r*), indicating how they were calculated

Our web collection on statistics for biologists contains articles on many of the points above.

Software and code

Policy information about availability of computer code

Data collection

SERS spectra of the samples were obtained using Renishaw in Via Confocal Raman Spectrometer Lei ca DMI 6000 epifluorescence microscope. 5 µl of buffy coat (blood) and 10 µl of cultured NK-92 cells were dropped on the OncoImmune probe platform. After 1 minute , Raman spectral scanning was done at 785 nm wavelength. Excitation wavelength of 785 nm was chosen for this experiment compatible for biological samples. Acquisition time per spectrum was 10 sec, repeated three times and averaged out, with laser power at SW. The spectra were collectively saved and processed using Spectragryph software (V1.2.9). The laser source was focused through a 20X objective on the samples.
Parameters for Renishaw in Via Raman microscope for this experiment were as follows -
- HC plan APO lenses with matched polarizer/ analyzer optics (magnification of 20X, N.A.0.7 0),
- Spot size was of 0. 625 µm radius
- Focal length of 250 mm
- Solid state laser
- Excitation wavelength 785nm (12.SmW)
- Matched polarizer optics power of lasers and
- Spectral resolution of 0.5 cm-1 in visible, 1 cm-1 in NUV and IR
- Spatial resolution:< 1 µm (lateral), <2µm (depth)

EzCAD2 software was used for laser fabrication.

Data analysis

Data analysis was done using commercially available PLS Toolbox software "SOLO" developed by Eigenvector Research Inc (version 9.0). For two tailed student t test graphpad Prism (version 9.2.0) was used . Other softwares like excel, wire 5.4 & spectragyrph (V1.2.9)were also

For manuscripts utilizing custom algorithms or software that are central to the research but not yet described in published literature, software must be made available to editors and reviewers. We strongly encourage code deposition in a community repository (e.g. GitHub). See the Nature Portfolio guidelines for submitting code & software for further information.

Data

Policy information about availability of data

All manuscripts must include a data availability statement. This statement should provide the following information, where applicable:

- Accession codes, unique identifiers, or web links for publicly available datasets
- A description of any restrictions on data availability
- For clinical datasets or third party data, please ensure that the statement adheres to our policy

Data supporting the findings of this study are available from the corresponding author upon reasonable request. The source data underlying figures 1(v), 2(ii-vi), 3 (B-i), 4(i), 5(A-E), 6(A, B, C), 7(A), 8(C), 9(C-D), Sup Fig2, 3,5(iii), 6B, 7A, 8A-B, 9A are provided as Source Data File.

Field-specific reporting

Please select the one below that is the best fit for your research. If you are not sure, read the appropriate sections before making your selection.

☒ Life sciences ☐ Behavioural & social sciences ☐ Ecological, evolutionary & environmental sciences

For a reference copy of the document with all sections, see nature.com/documents/nr-reporting-summary-flat.pdf

Life sciences study design

All studies must disclose on these points even when the disclosure is negative.

Sample size	Sample size was determined based on similar studies in this field. In in-vitro investigations, it was predetermined that a sample size of at least n=2 would allow for enough analysis to reach significant conclusions of the data due to the high reproducibility and uniformity between cell cultures. For usage of nonparametric statistical methods, we increased the sample number (n=32) for data validation.
Data exclusions	No data was excluded
Replication	All experiments were reproduced to reliably support conclusions stated in the manuscript. 1. For particle size calculation, 100 independent particles were measured and five independent experiments were performed. For gene expression analysis, three independent experiments were performed and and 7 independent experiments were performed for Raman. For diagnosis 20 biologically independent samples were used and Raman measurements were taken 10 times and averaged.
Randomization	Randomization was not relevant for our study since our study was taken with patients with known disease diagnosis.
Blinding	In our machine learning algorithm, the model was trained with training data from tumor associated NK cells and testing was done with patient buffy coat sample. Therefore the testing data was blind data which was not exposed to training algorithm.

Reporting for specific materials, systems and methods

We require information from authors about some types of materials, experimental systems and methods used in many studies. Here, indicate whether each material, system or method listed is relevant to your study. If you are not sure if a list item applies to your research, read the appropriate section before selecting a response.

Materials & experimental systems

n/a	Involved in the study	
☒	☐	Antibodies
☐	☒	Eukaryotic cell lines
☒	☐	Palaeontology and archaeology
☒	☐	Animals and other organisms
☐	☒	Human research participants
☒	☐	Clinical data
☒	☐	Dual use research of concern

Methods

n/a	Involved in the study	
☒	☐	ChIP-seq
☒	☐	Flow cytometry
☒	☐	MRI-based neuroimaging

Eukaryotic cell lines

Policy information about cell lines

Cell line source(s)	Cell lines- MDAMB231 (ATCC) , H69AR (ATCC), COLO 205 (ATCC), NK92 (ATCC) were purchased from American Type Culture Collection (ATCC), Primary NK cells (Stem cell technologies) , PBMC (Stem cell technologies)
Authentication	Cell lines were authenticated by ATCC & Stem cell Technologies by STR method prior to purchase.
Mycoplasma contamination	All cell lines were tested for mycoplasma contamination prior to purchase and all cell lines were tested negative for mycoplasma contamination.
Commonly misidentified lines (See ICLAC register)	None of the used cell lines is listed in ICLAC database.

Human research participants

Policy information about studies involving human research participants

Population characteristics	Blood samples of cancer patients (adult male and female) were obtained from Ontario Tumor Bank (OTB). OTB is a provincial bio bank that collects blood and tissue samples as well as personal health information (PHI) from consenting volunteer participants. OTB features direct control over every aspect of collection, storage and access to the inventory of cancer specimens with de-identified data. OTB access procedures ensure high ethical standards and protection of donor privacy and confidentiality.
Recruitment	Recruitment was undertaken by Ontario Tumor Bank from consenting volunteers. OTB is a provincial bio bank that collects blood and tissue samples as well as personal health information (PHI) from consenting volunteer participants. OTB features direct control over every aspect of collection, storage and access to the inventory of cancer specimens with de-identified data. OTB access procedures ensure high ethical standards and protection of donor privacy and confidentiality.
Ethics oversight	Research Ethics Board of Ryerson University REB (2020 – 275)

Note that full information on the approval of the study protocol must also be provided in the manuscript.
